# The rise in prevalence of autoantibodies neutralizing type I interferons reaches a plateau in centenarians

**DOI:** 10.70962/jhi.20260143

**Published:** 2026-10-27

**Authors:** Nanna Mørk, Julie A. Jensen, Paul Bastard, Lisbeth Aagaard Larsen, Bettina Bundgaard, Marianne Nygaard, Signe Bedsted Clemmensen, Chiaki Tao, Vibeke Sorensen Catts, Anne Puel, Guillaume Vogt, Ivan David Lozada-Martinez, Juan-Manuel Anaya, Yasumichi Arai, Perminder Singh Sachdev, Satoshi Okada, Jean-Laurent Casanova, Kaare Christensen, Aurélie Cobat, Trine H. Mogensen

**Affiliations:** 1 Department of Biomedicine, Aarhus University, Aarhus, Denmark; 2 Center for Immunology of Viral Infections, Aarhus University, Aarhus, Denmark; 3 Laboratory of Human Genetics of Infectious Diseases, Institut Imagine, Université Paris Cité, Necker Branch, INSERM UMR 1163, Paris, France; 4 Paris City University, Imagine Institute, Paris, France; 5 Pediatric Hematology-Immunology & Rheumatology Unit, Necker Hospital for Children, Assistance Publique-Hôpitaux de Paris, Paris, France; 6 Department of Public Health, Danish Aging Research Center, University of Southern Denmark, Odense, Denmark; 7 Department of Pediatrics, Hiroshima University Graduate School of Biomedical and Health Sciences, Hiroshima, Japan; 8 Discipline of Psychiatry & Mental Health School of Clinical Medicine, Faculty of Medicine and Health, Centre for Healthy Brain Ageing, UNSW Sydney, Sydney, Australia; 9 Department of Pediatrics, Necker Hospital for Sick Children, Assistance Publique-Hôpitaux de Paris, Paris, France; 10 Center for Healthy Longevity, Universidad de la Costa, Barranquilla, Colombia; 11 Colombian Centenarian Alliance, Bogotá, Colombia; 12 Center for Supercentenarian Medical Research, Keio University School of Medicine, Tokyo, Japan; 13 Human Biology Microbiome Quantum Research Center (Bio2Q), Keio University, Tokyo, Japan; 14 Laboratory of Human Genetics of Infectious Diseases, Center for the Genetics of Host Defense, University of Texas Southwestern, Dallas, TX, USA; 15 Departments of Pediatrics and Immunology, and Children Research Institute, University of Texas Southwestern, Dallas, TX, USA; 16 Howard Hughes Medical Institute, New York, NY, USA; 17 Department of Infectious Diseases, Aarhus University Hospital, Aarhus, Denmark

## Abstract

While autoantibodies (auto-Abs) neutralizing type I interferons (IFN-I) are relatively rare before the age of 65 (<1%), their prevalence reaches 2–3% between 70 and 80 years and >6% in individuals above 80 years in the general population. To assess the prevalence of IFN-I auto-Abs at the extreme end of human lifespan, we performed a multinational study of centenarians from four geographically and ethnically distinct cohorts collected in Denmark (*n* = 258), Colombia (*n* = 99), Australia (*n* = 281), and Japan (*n* = 300), comprising 938 individuals. Across cohorts, 51/938 centenarians (5.4%) harbored auto-Abs neutralizing IFN-I, ranging from 3.3% (Japan) to 9.1% (Colombia). We found that the prevalence of IFN-I auto-Abs levels off in centenarians compared with individuals ≥80 years from the general population, demonstrating that neutralizing IFN-I auto-Abs are not incompatible with longevity, despite conferring a risk of life-threatening viral infections. Yet, the plateau of their prevalence in centenarians suggests that they may not be beneficial for reaching exceptional longevity and may negatively impact mortality.

## Teaser

Mørk et al. investigated neutralizing IFN-I auto-Abs in centenarians from Denmark, Colombia, Australia, and Japan and found that the prevalence of auto-Abs reaches a plateau in centenarians and declines in Japanese and females, indicating that IFN-I auto-Abs can persist in centenarians across geographically and ethnically different populations, despite conferring a risk of life-threatening viral infections.

## Introduction

Human autoantibodies (auto-Abs) neutralizing type I interferons (IFN-I) were first reported in 1981 in a single case of severe disseminated varicella (1, 2). They were subsequently demonstrated to be present in patients with rare genetic diseases, such as defects in *AIRE* leading to autoimmune polyendocrine syndrome type 1 (3, 4), but were long thought to be clinically silent. The clinical significance of neutralizing IFN-I auto-Abs became evident during the COVID-19 pandemic, when these auto-Abs were associated with life-threatening disease. In particular, neutralizing IFN-I auto-Abs were shown to underlie 10–20% of severe and fatal cases of COVID-19 (5, 6, 7). IFN-I auto-Abs were later shown to underlie other severe viral diseases, including severe seasonal influenza A (8), West Nile virus (WNV) encephalitis (9, 10), fulminant viral hepatitis triggered by herpes simplex virus (11), and avian H5N1 influenza A virus (12), among others (13, 14, 15). Moreover, IFN-I auto-Abs were found to underlie adverse reactions to live-attenuated yellow fever (16) and chikungunya virus vaccines (17). In a large study of IFN-I auto-Abs in a French cohort of ∼34,000 individuals from the general population (6), the prevalence of auto-Abs neutralizing high concentrations (10 ng/ml) of IFN-α, -β, and -ω and low concentrations (100 pg/ml) of IFN-α and -ω under the age of 65 years was ∼0.5–1%, depending on the type and level of IFN-I (6, 18, 19), whereas the prevalence increased to ∼4–7% among individuals aged 80–85 years (6).

Subsequently, another study taking advantage of consecutive plasma samples from the Swiss HIV Cohort investigated the prevalence of neutralizing IFN-I auto-Abs in 1,876 individuals aged 65–94 years (20). This study reported an overall prevalence of neutralizing IFN-I auto-Abs of 1.2% for people living with HIV and on antiretroviral treatment, with a less pronounced age-related increase in this subpopulation, ranging from 0.6% in the 65–69-year-olds, increasing to 1.7% in the 70–79-year-olds, and a slight decrease to 1.6% in the 80–89-year-olds. This Swiss HIV Cohort study, together with other studies, also established an overall long-term persistence of neutralizing IFN-I auto-Abs, with detectable auto-Abs remaining for decades in some individuals. Moreover, the study suggested that viral infections or therapeutic IFN-I exposure may trigger the development of these auto-Abs in individuals with pre-existing defects in self-tolerance. Recently, a study demonstrated that in patients with severe COVID-19 and neutralizing IFN-I auto-Abs, circulating IFN-I–specific B cells can be detected prior to infection and acquire high affinity and neutralizing potential to IFN-I through extended somatic hypermutation, suggesting that germinal center–derived memory B cell responses directed against IFN-I are established before severe viral infection (21). These findings are consistent with the growing number of inborn errors of thymic negative selection underlying the production of these auto-Abs in children and young adults (4, 22, 23, 24, 25, 26). They suggest that age-related changes in immune regulation may promote the development or persistence of autoreactive antibodies targeting IFN-I pathways.

Existing population studies rarely include individuals at the extreme end of human lifespan, leaving age trajectories and the prevalence of IFN-I auto-Abs in oldest old individuals largely unknown. In this context, centenarians represent a unique demographic group that provides valuable insights into the biology of aging and immune resilience. Individuals who reach exceptional longevity often display distinctive genetic, physiological, and immunological characteristics compared with the general population (27, 28, 29). Studies of centenarian cohorts have revealed unusual patterns of immune function, including alterations in inflammatory regulation, immune cell composition, and antibody responses (30, 31). As a result, centenarians are frequently regarded as a natural model of successful ageing and a valuable population for investigating age-associated immune phenomena. At the same time, the immune profiles observed in these individuals may also reflect survivorship bias, whereby individuals with specific protective traits are more likely to reach advanced age. Finally, the prevalence is the result of the incidence of neutralizing IFN-I auto-Abs and the mortality associated with having neutralizing IFN-I auto-Abs. However, both of these are unknown among the oldest old. Importantly, it remains unclear whether the age-associated increase in IFN-I auto-Abs observed in older adults continues into the 10th and 11th decades of life, stabilizes, or declines among centenarians. Furthermore, the majority of available population-based studies have focused on single populations or limited geographic regions, leaving potential global variation insufficiently characterized (6, 20, 32). Understanding whether the prevalence of these auto-Abs differs between older populations may provide important insight into the relative contributions of genetic background, environmental exposures, and demographic factors associated with longevity. To address these questions, we conducted a multinational study investigating the prevalence of neutralizing IFN-I auto-Abs in centenarians from diverse geographic regions.

## Results

### Demographics and sample size of centenarian cohorts

**Figure 1.**
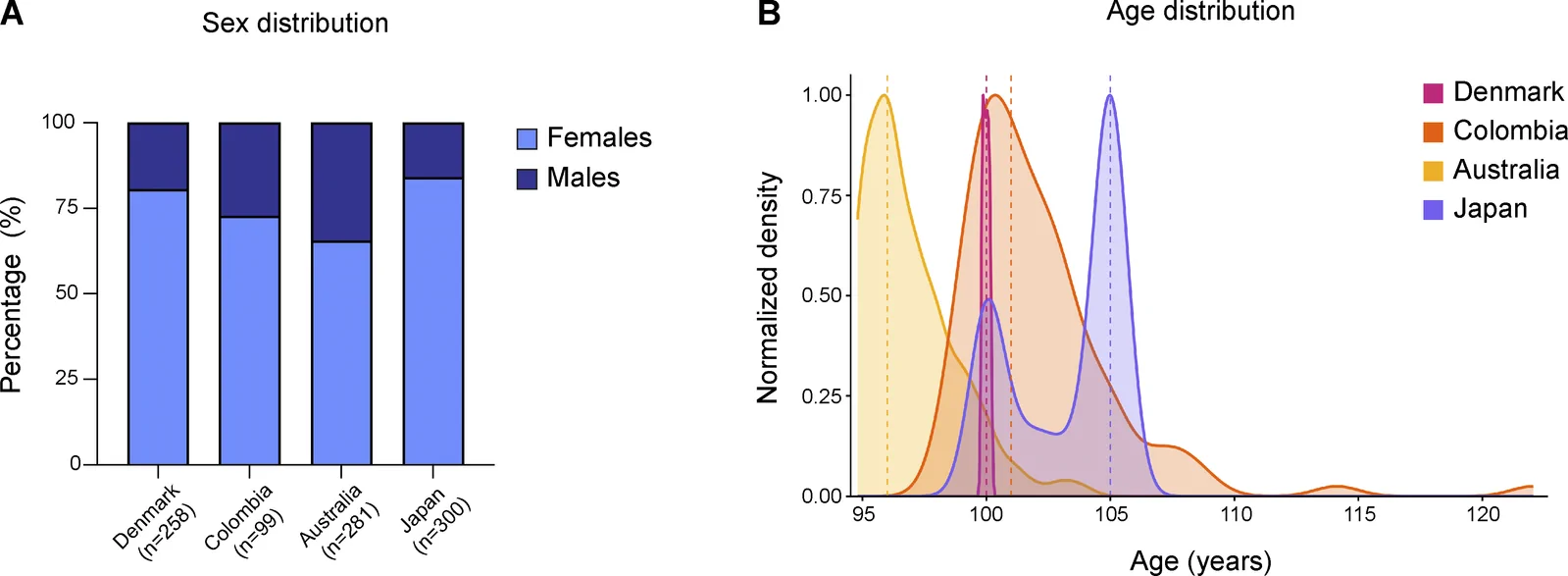
**Demographics of global centenarian cohorts. (A)** Sex distribution across centenarian cohorts from Denmark (*n* = 258), Colombia (*n* = 99), Australia (*n* = 281), and Japan (*n* = 300). Bars indicate the percentage of females and males within each cohort. **(B)** Age distribution of centenarian cohorts shown as kernel density plots with a jitter factor of 1. Density estimates were scaled for the maximum density within each cohort to equal 1. Dashed vertical lines indicate median ages for each cohort. In the Japanese cohort, ages recorded as “105+” were assigned an age of 105 years for analysis. Age data were available for all individuals in the Danish, Colombian, and Japanese cohorts, and were available for 279 of 281 individuals in the Australian cohort.

Plasma or serum samples were obtained from four independent cohorts from Denmark (*n* = 258), Colombia (*n* = 99), Australia (*n* = 281), and Japan (*n* = 300) (Table 1). The cohorts consisted of nonagenarians, centenarians, semi-supercentenarians, and a few supercentenarians and are collectively called centenarians throughout this study. Female predominance was observed across all cohorts, ranging from 65.5% in the Australian cohort to 84.0% in the Japanese cohort, with a combined prevalence across all cohorts of 76.3% (Fig. 1 A and Table 1). Although all cohorts were classified as centenarians, age distributions differed between the late 90s to 100 years or even above (Fig. 1 B and Table 1). All Danish individuals had their plasma sample taken at the age of 100 years; the Colombian cohort had a median age of 101 years (interquartile range [IQR], 100–103); the Australian cohort was younger overall (median 96 years, IQR, 96–98), whereas the Japanese cohort exhibited the highest median age (105 years, IQR, 100–105) (Fig. 1 B and Table 1). Ages above 105 in the Japanese cohort were recorded as “105+” and conservatively treated as 105 years for analysis.

**Table 1.** Summary and cohort characteristics, including total number of individuals, sex distribution, and median age with IQR

Cohort	Individuals (*N*)	Females, *n* (%)	Males, *n* (%)	Median age [IQR]
Denmark	258	208 (80.6)	50 (19.4)	100 [100–100]
Colombia	99	72 (72.7)	27 (27.3)	101 [100–103]
Australia	281	184 (65.5)	97 (34.5)	96 [96–98]
Japan	300	252 (84.0)	48 (16.0)	105 [100–105]
Combined	938	716 (76.3)	222 (23.7)	100 [99–102])

### Prevalence of neutralizing IFN-I auto-Abs in centenarians

**Figure 2.**
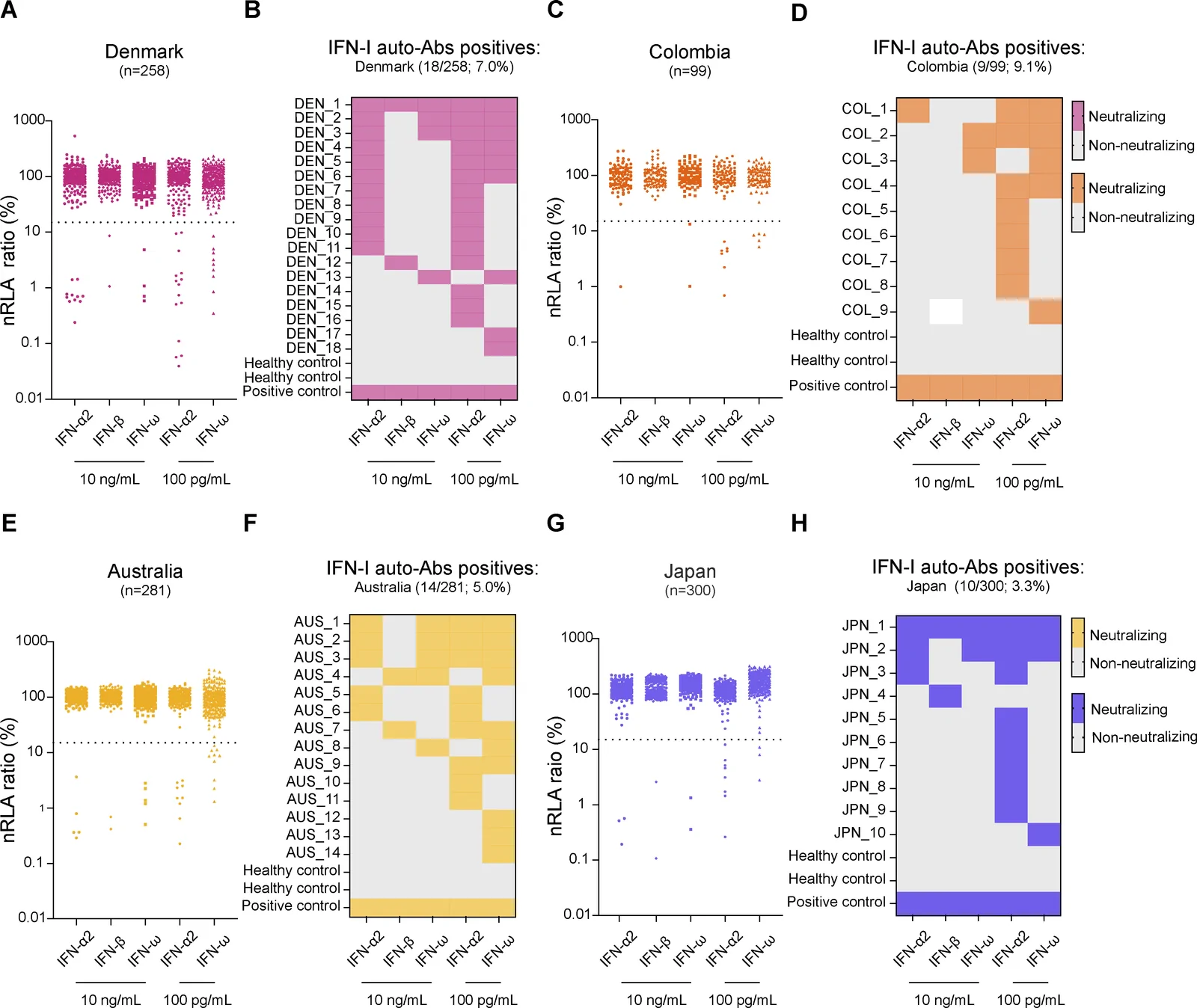
**Neutralizing IFN-I** **auto-Abs** **in global centenarian cohorts. (A, C, E, and G)** Neutralization of IFN-I signaling measured by luciferase-based HEK293T reporter assay in plasma or serum samples from centenarian cohorts from Denmark (A), Colombia (C), Australia (E), and Japan (G). RLA (nRLA, %) is shown following stimulation with IFN-α2, IFN-β, or IFN-ω at the indicated concentrations. Each dot represents one individual. Dashed horizontal lines indicate the predefined neutralization threshold (15% nRLA). **(B, D, F, and H)** Heatmaps summarizing subtype-specific IFN-I neutralization patterns among auto-Ab–positive Danish (B), Colombian (D), Australian (F), and Japanese (H) centenarians. Rows represent individual centenarians, and columns indicate IFN-I subtype and concentration tested. Colored squares indicate neutralizing activity, gray squares indicate non-neutralizing responses, and white indicates nonavailable samples. Healthy controls and positive controls are included at the bottom of each panel. Each sample was measured once at each IFN-I condition tested.

Neutralizing activity against IFN-I was assessed using a luciferase-based HEK293T reporter assay as previously described (6, 9). Unglycosylated IFN-α2 and IFN-ω were tested at 10 ng/ml and 100 pg/ml, and glycosylated IFN-β was tested at 10 ng/ml. Cohort-specific neutralization profiles are shown in Fig. 2, and subtype-specific neutralization patterns are summarized in Table 2. Among 258 Danish centenarians, a total of 18 individuals (7.0%) harbored neutralizing auto-Abs against at least one IFN-I subtype (Fig. 2, A and B). At 10 ng/ml, 13/258 (5.0%) demonstrated neutralizing activity, predominantly targeting IFN-α2 and/or IFN-ω (12/258, 4.7%), whereas IFN-β neutralization was less frequent (2/258, 0.8%). One individual neutralized all three IFN-I subtypes, and three were positive for more than one subtype at the concentration of 10 ng/ml. An additional 5/258 individuals (1.9%) displayed neutralization exclusively at 100 pg/ml, including three individuals neutralizing IFN-α2 and two neutralizing IFN-ω. In the Colombian cohort of 99 centenarians, nine individuals (9.1%) exhibited neutralizing activity against at least one IFN-I subtype (Fig. 2, C and D). At 10 ng/ml, neutralizing activity was observed in 3/99 individuals (3.0%) exclusively directed against IFN-α2 and/or IFN-ω, whereas no individuals neutralized IFN-β. No individuals neutralized more than one subtype at this concentration. The remaining 6/99 individuals (6.1%) demonstrated neutralization exclusively at 100 pg/ml, including five individuals neutralizing IFN-α2 and one individual neutralizing IFN-ω. Among the 281 Australian centenarians, 14 individuals (5.0%) exhibited neutralizing activity against at least one IFN-I subtype (Fig. 2, E and F). At 10 ng/ml, 8/281 individuals (2.8%) demonstrated neutralization, primarily directed against IFN-α2 and/or IFN-ω (7/281, 2.5%), whereas IFN-β neutralization was detected in 2/281 individuals (0.7%). Four individuals (1.4%) neutralized more than one subtype at this concentration. An additional 6/281 (2.1%) displayed neutralization exclusively at 100 pg/ml, including two individuals neutralizing IFN-α2, three neutralizing IFN-ω, and one individual neutralizing both IFN-α2 and IFN-ω at this concentration. Finally, among the 300 Japanese centenarians, 10 individuals (3.3%) exhibited neutralizing anti–IFN-I auto-Abs (Fig. 2, G and H). At 10 ng/ml, neutralizing activity was detected in 4/300 individuals (1.3%), including 3/300 (1.0%) targeting IFN-α2 and/or IFN-ω and 1/300 (0.3%) targeting IFN-β. One individual neutralized all three IFN-I subtypes, and 2/300 (0.7%) neutralized more than one subtype at this concentration. An additional 6/300 (2.0%) individuals exhibited neutralization exclusively at 100 pg/ml, predominantly against IFN-α2 (5/300, 1.7%), whereas one individual neutralized IFN-ω only. Across all cohorts, individuals positive at 10 ng/ml also exhibited neutralizing activity at 100 pg/ml. Collectively, the prevalence of neutralizing IFN-I auto-Abs ranged from 3.3% in the Japanese cohort to 9.1% in the Colombian cohort, although no statistically significant difference in prevalence was observed across cohorts (Pearson’s χ^2^ test, χ^2^ = 6.46; degrees of freedom = 3; P = 0.091). When combining all four cohorts, 51 of 938 centenarians (5.4%) exhibited neutralizing auto-Abs against at least one IFN-I subtype.

**Table 2.** Prevalence of centenarians with IFN-I–neutralizing auto-Abs per IFN-I subtype and concentration tested

Cohort	IFN-α2 10 ng/ml	IFN-β 10 ng/ml	IFN-ω 10 ng/ml	IFN-α2 100 pg/ml	IFN-ω 100 pg/ml	Any IFN-I
Denmark *n/N* (%)	11/256^a^ (4.3)	2/258 (0.8)	4/258 (1.6)	15/258 (5.8)	9/257^a^ (3.5)	18/258 (7.0)
Colombia *n/N* (%)	1/98^a^ (1.0)	0/98^a^ (0.0)	2/99 (2.0)	7/99 (7.1)	5/99 (6.1)	9/99 (9.1)
Australia *n/N* (%)	5/279^a^ (1.8)	2/280^a^ (0.7)	5/280^a^ (1.8)	9/281 (3.2)	9/279^a^ (3.2)	14/281 (5.0)
Japan *n/N* (%)	3/300 (1.0)	2/300 (0.7)	2/300 (0.7)	8/300 (2.7)	3/300 (1.0)	10/300 (3.3)
Combined *n/N* (%)	20/933 (2.1)	6/936 (0.6)	13/937 (1.4)	39/938 (4.2)	26/935 (2.8)	51/938 (5.4)

n/N, number positive/number tested.

a Samples excluded due to inefficient transfection efficacy in HEK293T cell luciferase assay.

### Prevalence of neutralizing IFN-I auto-Abs in centenarians stratified by sex

The prevalence of neutralizing IFN-I auto-Abs between females and males across all centenarian cohorts and within each centenarian cohort was compared to investigate whether prevalence differed by sex (Table 3). The prevalence of neutralizing IFN-I auto-Abs was comparable between males and females across all cohorts, both separately and combined, with no statistically significant sex-associated differences observed (Danish: [P = 0.76], Colombian: [P = 1], Australian: [P = 0.25], and Japanese: [P = 1]). In the combined analysis across all cohorts (*n* = 938), 14/222 males (6.3%) and 37/716 females (5.2%) were positive for neutralizing IFN-I auto-Abs. The overall odds ratio (OR) for centenarian males versus centenarian females was 1.29 (95% confidence interval [CI], 0.63–2.50; P = 0.49), indicating no significant association between sex and the presence of neutralizing IFN-I auto-Abs.

**Table 3.** Prevalence of neutralizing IFN-I auto-Abs stratified by sex across centenarian cohorts

Cohort	Individuals (*N*)	Males, *n/N* (%)	Females, *n/N* (%)	OR (95% CI)	P value
Denmark	258	4/50 (8.0)	14/208 (6.7)	1.20 (0.28–4.08)	P = 0.76
Colombia	99	2/27 (7.4)	7/72 (9.7)	0.74 (0.07–4.28)	P = 1
Australia	281	7/97 (7.2)	7/184 (3.8)	1.96 (0.57–6.78)	P = 0.25
Japan	300	1/48 (2.1)	9/252 (3.6)	0.57 (0.01–4.33)	P = 1
Combined	938	14/222 (6.3)	37/716 (5.2)	1.29 (0.63–2.50)	P = 0.49

n/N, number positive/number tested. ORs for males relative to females with corresponding 95% CIs and P values are shown. Statistical comparisons were performed using Fisher’s exact test. Data are shown as numbers of individuals with auto-Abs relative to the total number within each sex (*n/N*, %).

### Comparison of neutralizing IFN-I auto-Abs prevalence in centenarians to the French general population

**Figure 3.**
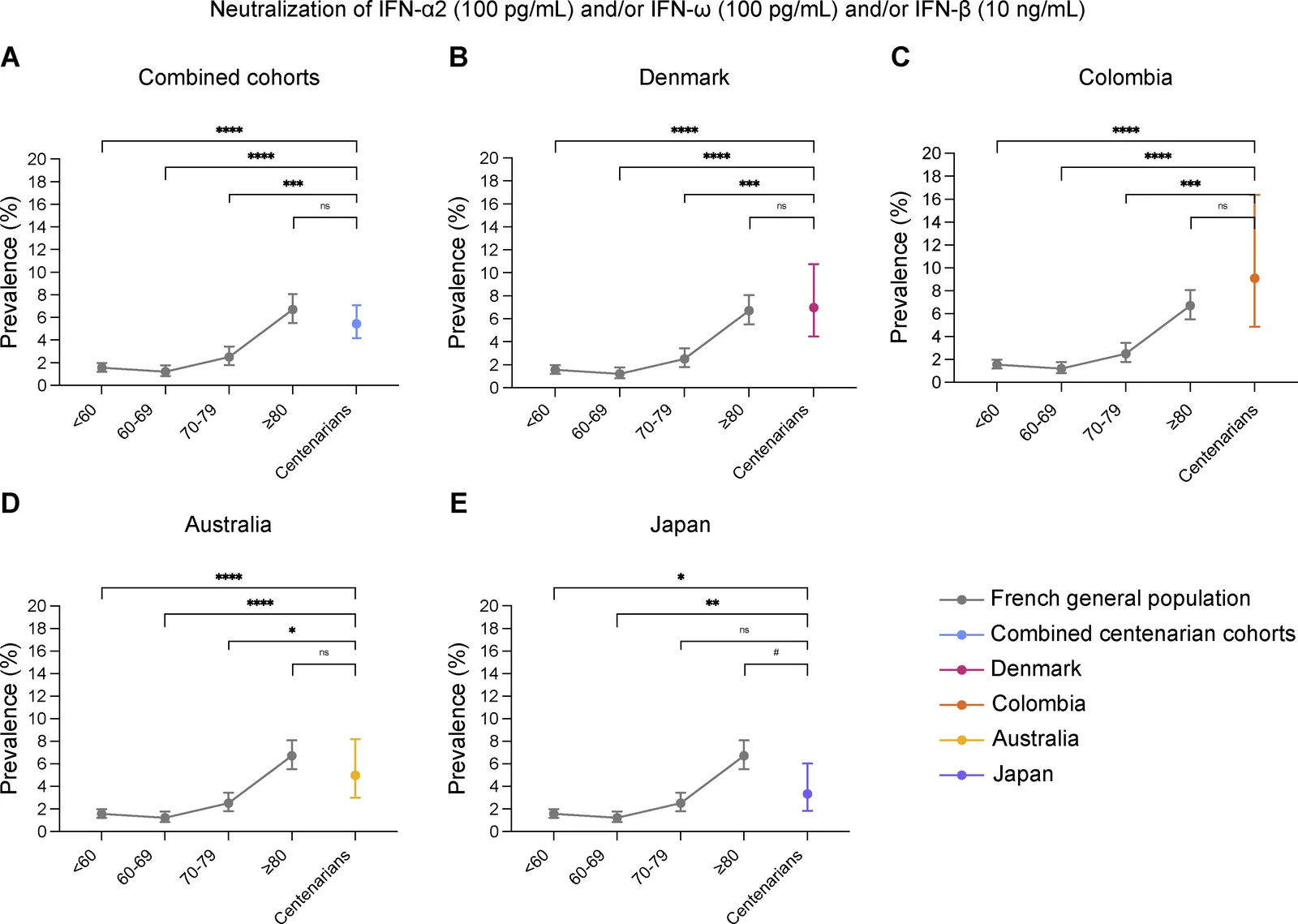
**Comparison of IFN-I–neutralizing** **auto-Abs** **prevalence between centenarian cohorts and age-stratified individuals from the French general population.** Prevalence of neutralizing auto-Abs against IFN-α2 and/or IFN-ω at 100 pg/ml and/or IFN-β at 10 ng/ml in centenarian cohorts compared with age-stratified individuals from a previously published French population cohort representative of the general population. French population data were derived from Bastard et al. and Manry et al. and included individuals grouped as <60 years (*N* = 4,128), 60–69 years (*N* = 2,164), 70–79 years (*N* = 1,443), and ≥80 years (*N* = 1,549) of age (6, 18). **(A)** Combined centenarian cohorts (Denmark, Colombia, Australia, and Japan). **(B–E)** Danish (B), Colombian (C), Australian (D), and Japanese (E) centenarian cohorts analyzed separately. Colored points represent prevalence estimates for each centenarian cohort, whereas gray points and lines represent prevalence estimates across the French age groups. Error bars indicate 95% CIs calculated using the Wilson method. ORs, 95% CIs, and P values were calculated by comparing each centenarian cohort to the corresponding French age groups using chi-squared or Fisher’s exact test as appropriate. Statistical significance is indicated as follows: ns, not significant; #P < 0.05; *P < 0.05; **P < 0.01; ***P < 0.001; ****P < 0.0001.

**Figure 4.**
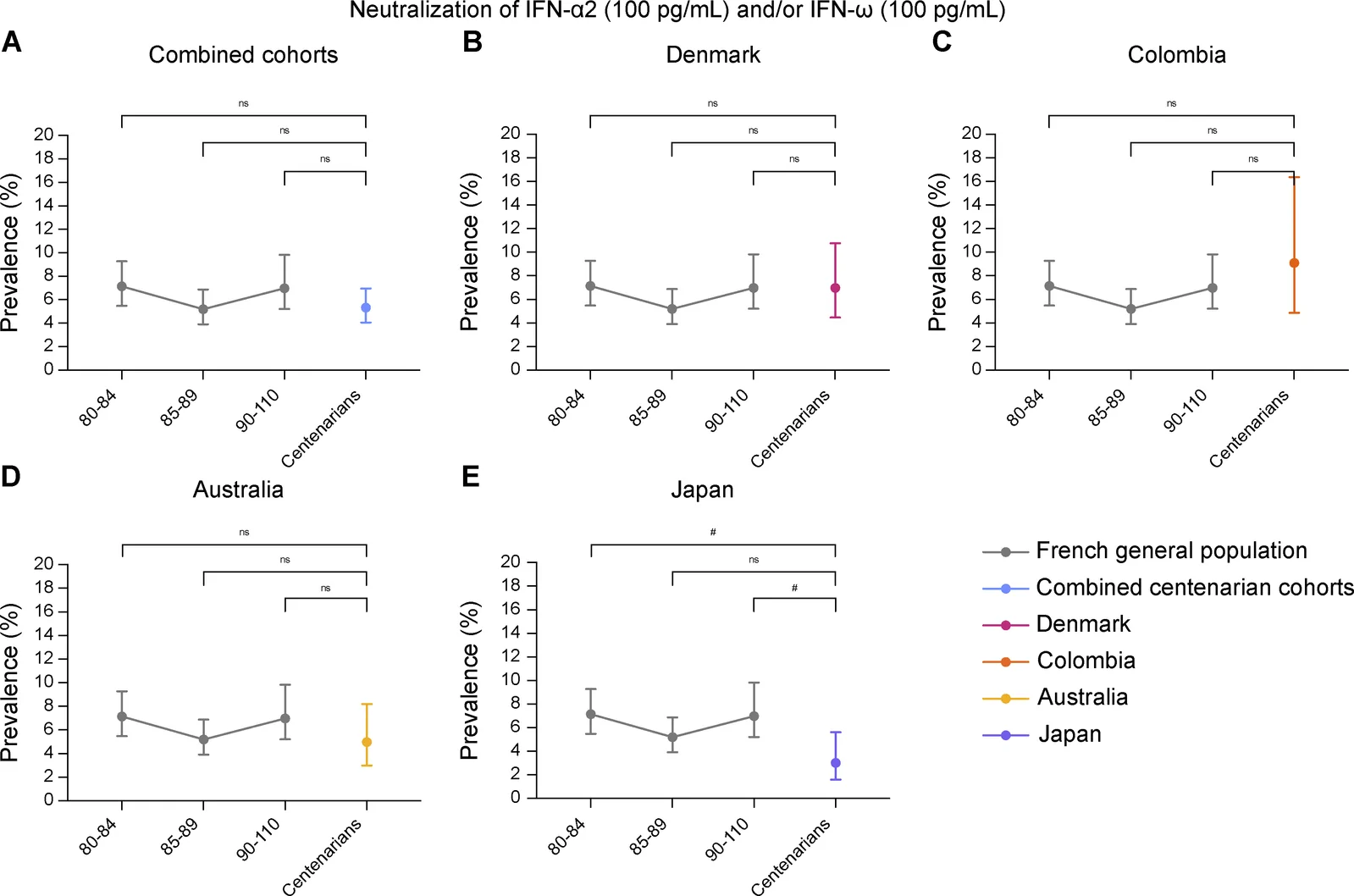
**Comparison of IFN-I–neutralizing** **auto-Abs** **prevalence between centenarian cohorts and age-stratified individuals among the oldest from the French general population.** Prevalence of neutralizing auto-Abs against IFN-α2 and/or IFN-ω at 100 pg/ml in centenarian cohorts compared with age-stratified individuals from a previously published French population cohort representative of the general population. French population data were derived from Bastard et al. and included individuals grouped as <80–84 years (*N* = 714), 85–89 years (*N* = 866), and 90–110 years (*N* = 487) of age (6, 18). **(A)** Combined centenarian cohorts (Denmark, Colombia, Australia, and Japan). **(B–E)** Danish (B), Colombian (C), Australian (D), and Japanese (E) centenarian cohorts analyzed separately. Colored points represent prevalence estimates for each centenarian cohort, whereas gray points and lines represent prevalence estimates across the French population age groups. Error bars indicate 95% CIs calculated using the Wilson method. ORs, 95% CIs, and P values were calculated by comparing each centenarian cohort to the corresponding French population age groups using chi-squared or Fisher’s exact test as appropriate. Statistical significance is indicated as follows: ns, not significant; #P < 0.05.

We compared the prevalence of neutralizing IFN-I auto-Abs in the centenarian cohorts with that observed across older adult age groups in a large French general population-based cohort (6, 18). Complete statistical results for all comparisons, including ORs, 95% CIs, and P values, are provided in Tables S1, S2, S3, S4, S5, and S6. Across all four centenarian cohorts combined, the prevalence of neutralizing IFN-I auto-Abs was similar to that observed in individuals aged ≥80 years from the French general population (Fig. 3 A and Table S1). In contrast, centenarians exhibited a significantly higher prevalence than individuals aged <60 years (P < 0.0001), 60–69 years (P < 0.0001), and 70–79 years (P = 0.0001), as expected. When analyzing the centenarian cohorts separately, modest geographical differences in IFN-I auto-Abs prevalence were observed. Nevertheless, the Danish (Fig. 3 B), Colombian (Fig. 3 C), and Australian (Fig. 3 D) cohorts showed the same overall pattern as the combined cohort, with no significant difference in the prevalence compared with the French individuals aged ≥80 years, but significantly higher prevalences than those observed in individuals aged <60, 60–69, and 70–79 years (Table S1). Notably, the Japanese cohort exhibited the lowest prevalence of neutralizing IFN-I auto-Abs, which was significantly lower than that for the French individuals aged ≥80 years (P = 0.047) (Fig. 3 E and Table S1). No significant difference was observed compared with the 70–79-year age group (P = 0.37). Consistent with the other centenarian cohorts, however, prevalence remained significantly higher than in individuals aged <60 years (P = 0.020) and 60–69 years (P = 0.0039) (Table S1). In addition, we stratified the ≥80 age group from the French general population into smaller strata with the age groups 80–84, 85–89, and 90–110 years and compared the prevalence of IFN auto-abs against IFN-α2 and/or IFN-ω (100 pg/ml) with the centenarian cohorts, both combined and separately (Fig. 4 and Table S2). No significant difference was observed between the centenarian cohorts combined and the oldest age groups from the French general population, supporting the data showing that the prevalence of IFN-I auto-Abs reaches a plateau, rather than continuing to increase, at the extreme end of human lifespan.

### Sex-stratified prevalence of neutralizing IFN-I auto-Abs in centenarians compared with the French general population

**Figure S1.**
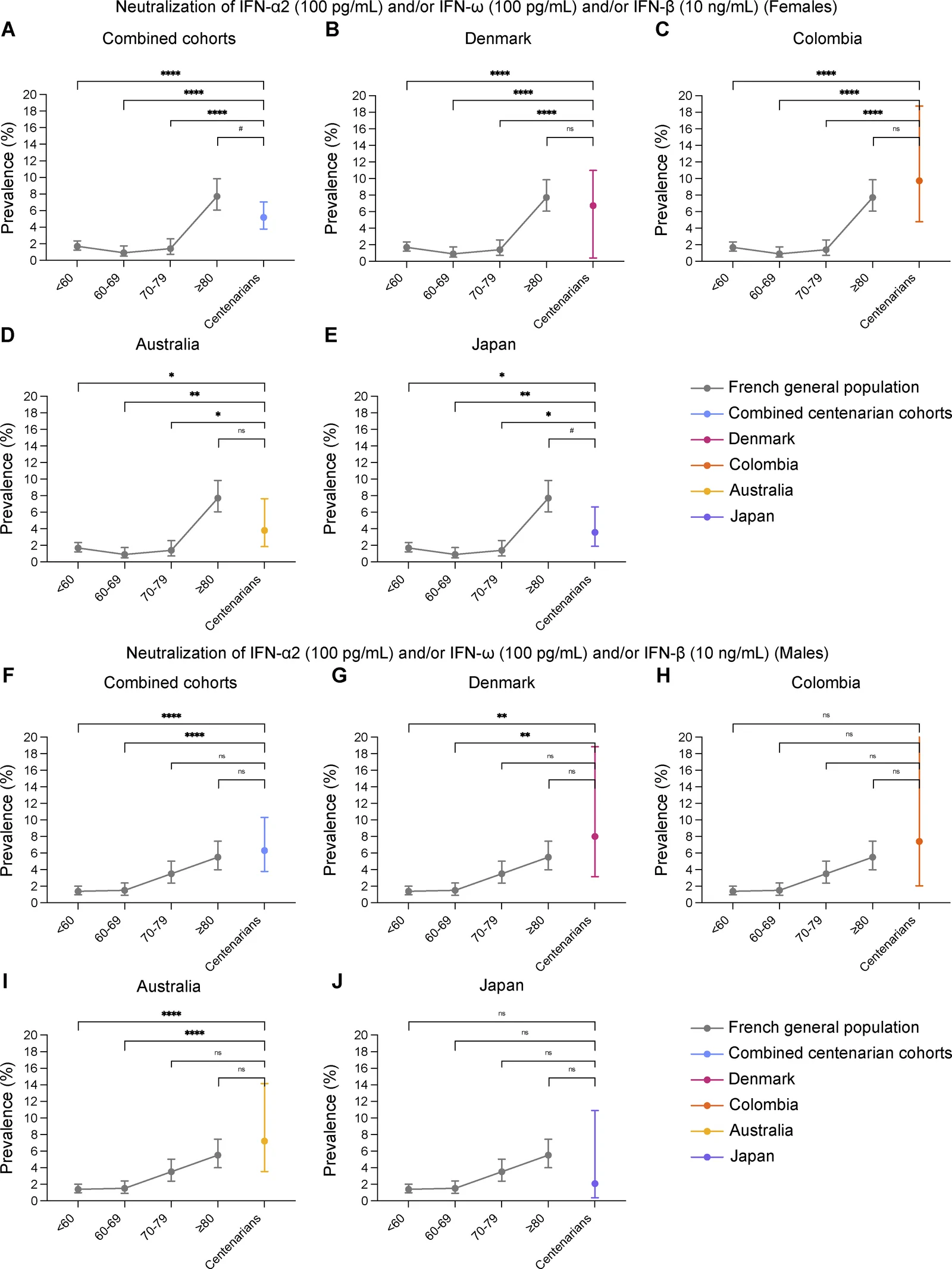
**Sex-stratified comparison of IFN-I–neutralizing** **auto-Abs** **prevalence between centenarian cohorts and age-stratified individuals from the French general population.** Prevalence of neutralizing auto-Abs against IFN-α2 and/or IFN-ω at 100 pg/ml and/or IFN-β at 10 ng/ml in centenarian cohorts compared with age-stratified individuals from a previously published French population cohort representative of the general population. French population data were derived from Bastard et al. and Manry et al. and included individuals grouped as <60 years (*N* = 2,122 for females and *N* = 2,015 for males), 60–69 years (*N* = 1,054 for females and *N* = 1,084 for males), 70–79 years (*N* = 656 for females and *N* = 752 for males), and ≥80 years (*N* = 775 for females and *N* = 677 for males) of age (6, 18). **(A–E)** Female centenarians. **(A)** Combined female centenarian cohorts (Denmark, Colombia, Australia, and Japan). **(B–E)** Danish (B), Colombian (C), Australian (D), and Japanese (E) female centenarian cohorts analyzed separately. **(F–J)** Male centenarians. **(F)** Combined male centenarian cohorts (Denmark, Colombia, Australia, and Japan). **(G–J)** Danish (G), Colombian (H), Australian (I), and Japanese (J) male centenarian cohorts analyzed separately. Colored points represent prevalence estimates for each centenarian cohort, whereas gray points and lines represent prevalence estimates across the French age groups. Error bars indicate 95% CIs calculated using the Wilson method. ORs, 95% CIs, and P values were calculated by comparing each centenarian cohort to the corresponding French age groups using chi-squared or Fisher’s exact test, as appropriate. Statistical significance is indicated as follows: ns, not significant; #P < 0.05;*P < 0.05; **P < 0.01; ****P < 0.0001.

Because the centenarian cohorts were predominantly female, both individually and when combined (Table 1), whereas the French general population cohort has an equal distribution of males and females (6), we next compared the prevalence of neutralizing IFN-I auto-Abs with the French general population after stratification by sex (Fig. S1, A–J). Among females (Fig. S1, A–E), the combined female centenarian cohort exhibited a significantly lower prevalence of neutralizing IFN-I auto-Abs than the females from the French population aged ≥80 years (P = 0.044) (Fig. S1 A and Table S3). Geographical stratification demonstrated that this difference was primarily driven by the Japanese female cohort (P = 0.016) (Fig. S1 E). Both the combined female centenarian cohort and each of the individual geographical centenarian cohorts exhibited significantly higher prevalences than French females aged <60 years, 60–69 years, and 70–79 years (Fig. S1, A–E and Table S3). Similar patterns were observed among the centenarian male cohort compared with males from the French general population (Fig. S1, F–J), although statistical significance was reached less consistently and not for centenarian males compared with males ≥80 years in the French population (Table S3). Overall, sex stratification did not alter the principal findings of a plateau in IFN-I auto-Abs prevalence in centenarians compared with the older general population, although the statistics for males were limited by the substantially smaller number of male centenarians available for analysis.

### Subtype-specific neutralizing IFN-I auto-Abs prevalence compared with the French general population

**Figure S2.**
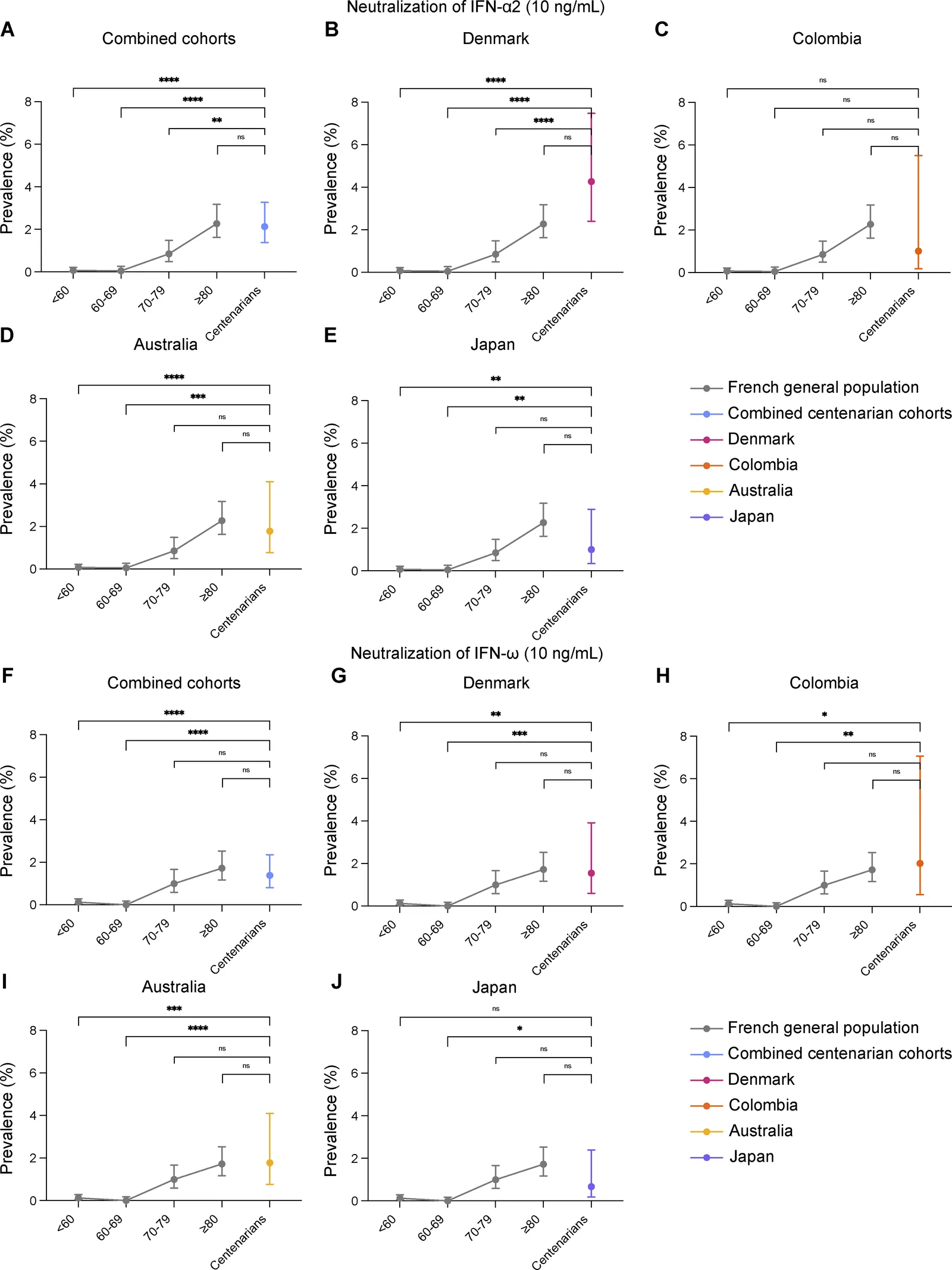
**Comparison of IFN-I–neutralizing** **auto-Abs** **prevalence between centenarian cohorts and age-stratified individuals from the French general population at high concentrations of IFN-α2 and IFN-ω.** Prevalence of neutralizing auto-Abs against IFN-α2 at 10 ng/ml or against IFN-ω at 10 ng/ml in centenarian cohorts compared with age-stratified individuals from a previously published French population cohort representative of the general population. French population data were derived from Bastard et al. and Manry et al. and included individuals grouped as <60 years (*N* = 4,128), 60–69 years (*N* = 2,138), 70–79 years (*N* = 1,408), and ≥80 years (*N* = 1,452) of age (6, 18). **(A)** IFN-α2 10 ng/ml combined centenarian cohorts (Denmark, Colombia, Australia, and Japan). **(B–E)** Danish (B), Colombian (C), Australian (D), and Japanese (E) centenarian cohorts analyzed separately. **(F)** IFN-ω 10 ng/ml combined centenarian cohorts (Denmark, Colombia, Australia, and Japan). **(G–J) **Danish (G), Colombian (H), Australian (I), and Japanese (J) centenarian cohorts analyzed separately. Colored points represent prevalence estimates for each centenarian cohort, whereas gray points and lines represent prevalence estimates across the French age groups. Error bars indicate 95% CIs calculated using the Wilson method. ORs, 95% CIs, and P values were calculated by comparing each centenarian cohort to the corresponding French age groups using chi-squared or Fisher’s exact test, as appropriate. Statistical significance is indicated as follows: ns, not significant; *P < 0.05; **P < 0.01; ***P < 0.001; ****P < 0.0001.

**Figure S3.**
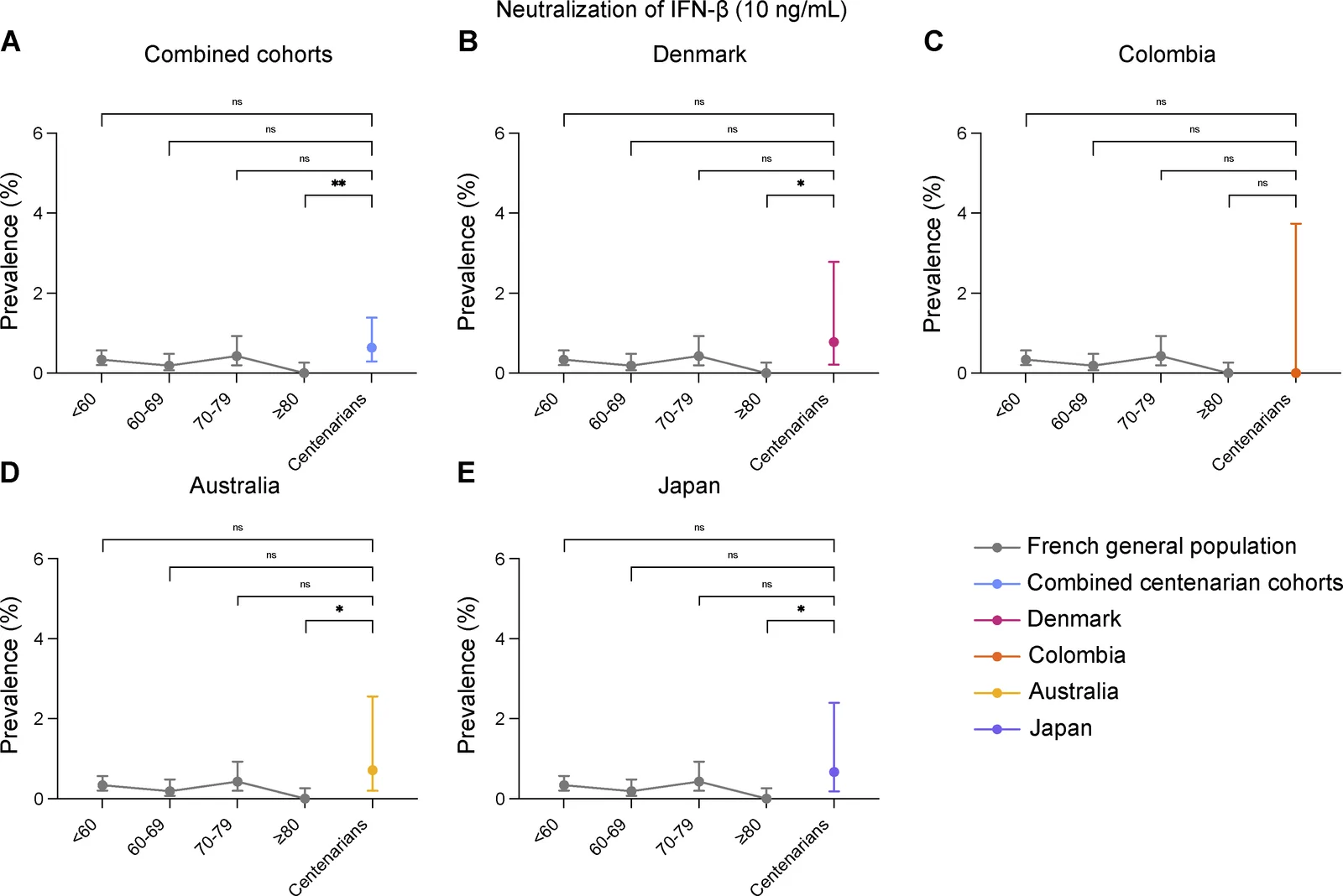
**Comparison of IFN-β–neutralizing** **auto-Abs** **prevalence between centenarian cohorts and age-stratified individuals from the French general population.** Prevalence of neutralizing auto-Abs against IFN-β at 10 ng/ml in centenarian cohorts compared with age-stratified individuals from a previously published French population cohort representative of the general population. French population data were derived from Bastard et al. and Manry et al. and included individuals grouped as <60 years (*N* = 4,114), 60–69 years (*N* = 2,138), 70–79 years (*N* = 1,408), and ≥80 years (*N* = 1,452) of age (6, 18). **(A)** IFN-β 10 ng/ml combined centenarian cohorts (Denmark, Colombia, Australia, and Japan). **(B–E)** Danish (B), Colombian (C), Australian (D), and Japanese (E) centenarian cohorts analyzed separately. Colored points represent prevalence estimates for each centenarian cohort, whereas gray points and lines represent prevalence estimates across the French age groups. Error bars indicate 95% CIs calculated using the Wilson method. ORs, 95% CIs, and P values were calculated by comparing each centenarian cohort with the corresponding French age groups using chi-squared or Fisher’s exact test, as appropriate. Statistical significance is indicated as follows: ns, not significant; *P < 0.05; **P < 0.01.

**Figure S4.**
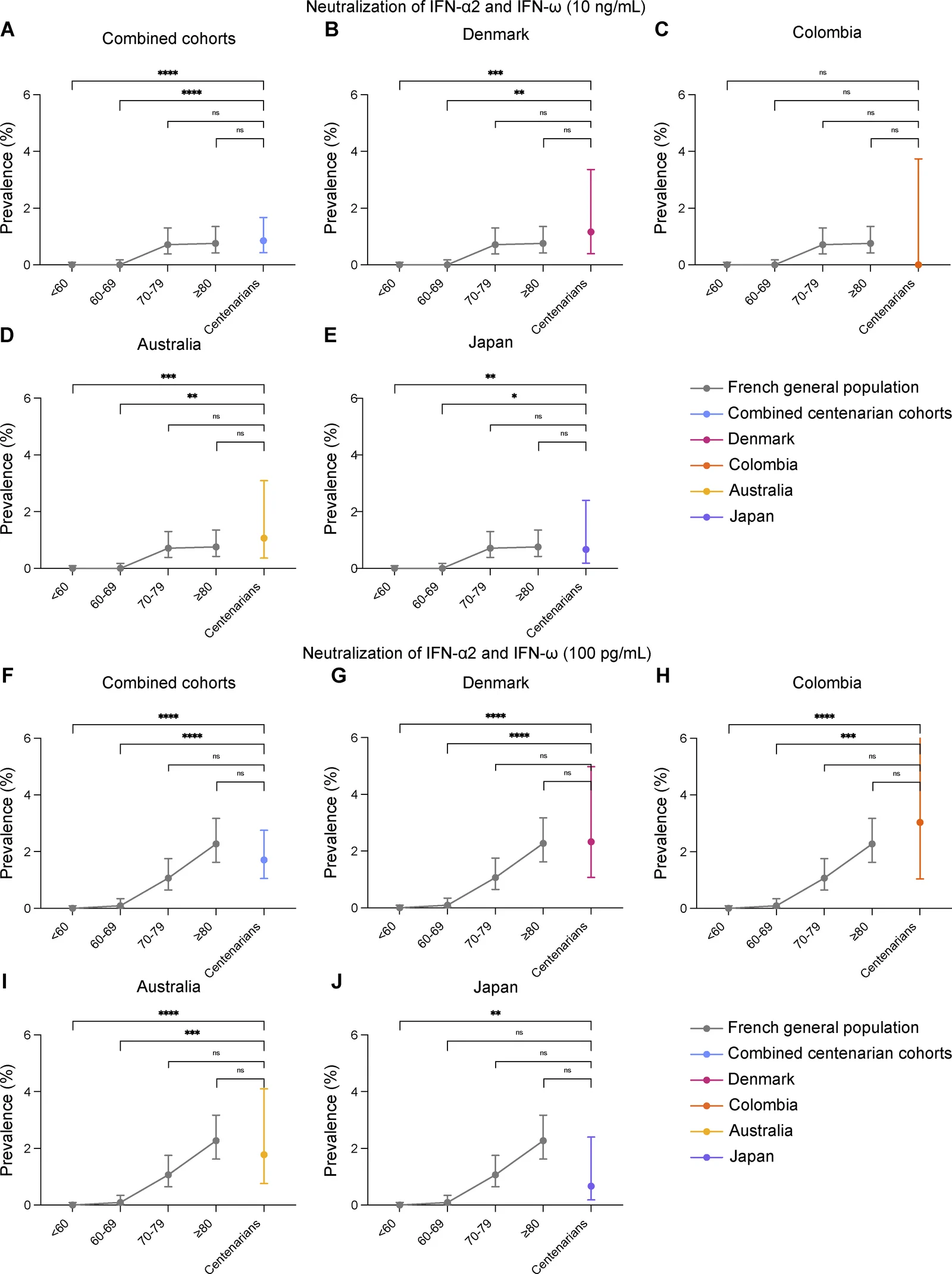
**Comparison of IFN-I–neutralizing** **auto-Abs** **prevalence between centenarian cohorts and age-stratified individuals from the French general population under IFN-α and IFN-ω double-positive conditions.** Prevalence of neutralizing auto-Abs against IFN-α2 and IFN-ω at high (10 ng/ml) or low (100 pg/ml) concentrations in centenarian cohorts compared with age-stratified individuals from a previously published French population cohort representative of the general population. French population data were derived from Bastard et al. and Manry et al. and included individuals grouped as <60 years (*N* = 4,128), 60–69 years (*N* = 2,138), 70–79 years (*N* = 1,408), and ≥80 years (*N* = 1,452) of age (6, 18). **(A)** IFN-α2 and IFN-ω 10 ng/ml combined centenarian cohorts (Denmark, Colombia, Australia, and Japan). **(B–E)** Danish (B), Colombian (C), Australian (D), and Japanese (E) centenarian cohorts analyzed separately. **(F)** IFN-α and IFN-ω 100 pg/ml combined centenarian cohorts (Denmark, Colombia, Australia, and Japan). **(G–J)** Danish (G), Colombian (H), Australian (I), and Japanese (J) centenarian cohorts analyzed separately. Colored points represent prevalence estimates for each centenarian cohort, whereas gray points and lines represent prevalence estimates across the French age groups. Error bars indicate 95% CIs calculated using the Wilson method. ORs, 95% CIs, and P values were calculated by comparing each centenarian cohort to the corresponding French age groups using chi-squared or Fisher’s exact test, as appropriate. Statistical significance is indicated as follows: ns, not significant; *P < 0.05; **P < 0.01; ***P < 0.001; ****P < 0.0001.

Next, we investigated whether centenarians differed from the French general population with respect to (1) the titer of IFN-I neutralization and (2) the breadth of IFN-I subtype neutralization. To address the first question, we examined subtype-specific neutralization patterns at high IFN-I concentrations (10 ng/ml) by comparing the centenarian cohorts with the age-stratified French population cohort (Fig. S2). For neutralization against IFN-α2 or IFN-ω, the overall pattern mirrored the observations for combined IFN-I neutralization (see section: Comparison of neutralizing IFN-I auto-Abs prevalence in centenarians to the French general population). Across the combined centenarian cohort, as well as the Danish, Australian, and Japanese cohorts, the rise in IFN-I auto-Abs over age reached a plateau and was not significantly higher than that of individuals aged ≥80 years. As expected, the IFN-I auto-Abs prevalence was significantly higher in centenarians than in individuals aged <60 years and 60–69 years. The Colombian cohort showed the same overall trend, although comparisons did not consistently reach statistical significance (Fig. S2, A–J, and Table S4). In contrast, neutralization against IFN-β at 10 ng/ml displayed a distinct pattern (Fig. S3, A–E). Previous studies have reported that the prevalence of IFN-β neutralizing auto-Abs does not increase substantially with age (6). Consistent with these findings, the prevalence of IFN-β–neutralizing auto-Abs in the centenarian cohorts was generally comparable with that observed in the French individuals younger than 80 years. However, all centenarian cohorts, except the Colombian cohort, exhibited a higher prevalence of IFN-β neutralization than the French population aged ≥80 years (Table S5). Finally, to address the second question regarding the breadth of IFN-I–neutralizing auto-Abs, we investigated whether centenarians exhibited broader IFN-I neutralization, defined as simultaneous neutralization of both IFN-α2 and IFN-ω (“double-positive” individuals) (Fig. S4). Overall, the same pattern was observed as for combined IFN-I neutralization. Across most centenarian cohorts, the prevalence of double-positive individuals was significantly higher than in the French population <60-year and 60–69-year age groups, whereas no significant differences were observed when comparing centenarians with the 70–79-year and ≥80-year age groups (Fig. S4, A–J, and Table S6).

### Age distribution of neutralizing IFN-I auto-Ab–positive centenarians

**Figure S5.**
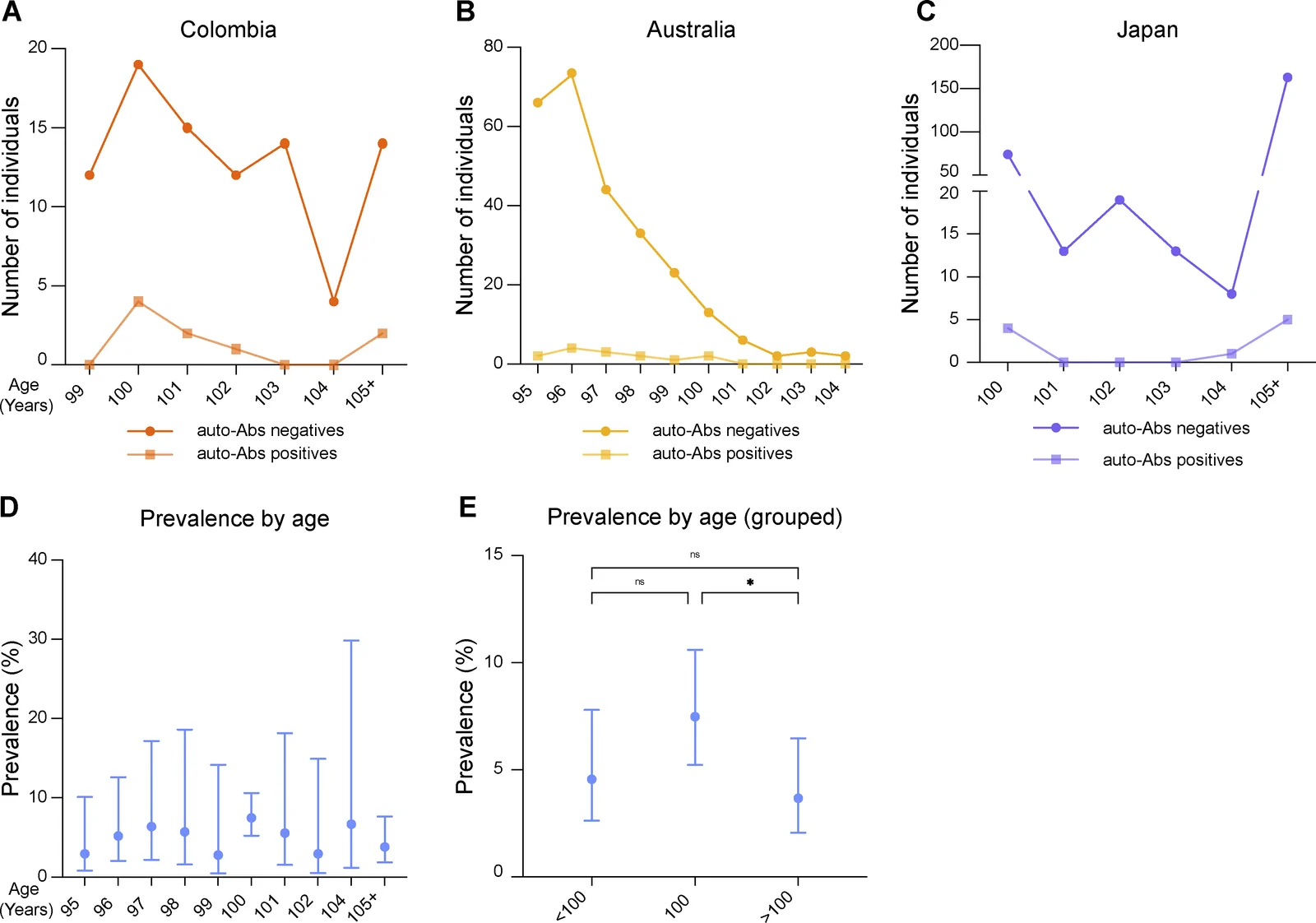
**Prevalence of neutralizing IFN-I** **auto-Abs** **according to age within the centenarian cohorts. (A–C)** Distribution of individuals positive and negative for neutralizing IFN-I auto-Abs according to age in the Colombian (A), Australian (B), and Japanese (C) centenarian cohorts. **(D)** Prevalence of neutralizing IFN-I auto-Abs across individual age groups (95–105+ years) in the combined centenarian cohorts (Denmark, Colombia, Australia, and Japan). Points represent prevalence estimates and error bars indicate 95% CIs calculated using the Wilson method. **(E)** Prevalence of neutralizing IFN-I auto-Abs in the combined centenarian cohorts after grouping individuals into those <100 years, exactly 100 years, and >100 years. Statistical comparisons were performed using a chi-squared test. Statistical significance is indicated as follows: ns, not significant; *P < 0.05.

Since the ages in all centenarian cohorts, except the Danish, extended beyond 100 years (Fig. 1 B), we next investigated whether the prevalence of neutralizing IFN-I auto-Abs varied across age groups within these centenarian cohorts (Fig. S5). In the Colombian (Fig. S5 A) and the Japanese (Fig. S5 C) cohorts, there was a peak in IFN-I auto-Ab–positive individuals in both the 100-year-olds as well as the 105+-year-olds, though ages were pooled in the 105+ groups, which could explain this peak. The IFN-I auto-Ab–positive individuals were evenly distributed across ages in the Australian centenarians (Fig. S5 B). To further assess age-associated trends, we combined all cohorts and compared the prevalence of IFN-I auto-Abs across age groups from 95 years with 105+ years (Fig. S5 D). No differences in prevalence were observed between age groups, suggesting that the prevalence of neutralizing IFN-I auto-Abs remains relatively stable across ages within the centenarian range. However, when age was grouped into individuals aged <100 years, exactly 100 years, and >100 years, a modest but significant reduction in prevalence was observed among individuals at ≥100 years compared with those aged exactly 100 years (P = 0.036), whereas the other pairwise comparisons did not reach significance (Fig. S5 E).

### Association between neutralizing IFN-I auto-Abs and history of infection, autoimmunity, and cancer reported by questionnaires

The association between neutralizing IFN-I auto-Abs and clinical conditions in which IFN-I are known to play important immunological roles, including infections, autoimmune disease, and cancer, was assessed (Table 4). Information on these conditions was available for the Danish, Colombian, and Japanese cohorts obtained through questionnaires or medical interviews. In Danish centenarians, there was no significant difference in autoimmune disease (33.3% vs. 20%, P = 0.32) or cancer (33.3% vs. 17.4%, P = 0.16) in the IFN-I auto-Ab–positive individuals compared with the IFN-I auto-Ab–negative individuals. In the Colombian centenarians, no differences were observed for autoimmune disease (11.1% vs. 8.9%, P = 0.59) or infection history (11.1% vs. 16.7%, P = 1) for the IFN-I auto-Ab–positive individuals compared with the IFN-I auto-Ab–negative individuals, and also no significant differences were observed for cancer, although this was more common among IFN-I auto-Ab–positive individuals compared with IFN-I auto-Ab–negative individuals (22.2% vs. 4.4%, P = 0.09). In the Japanese centenarians, cancer prevalence was largely identical between auto-Ab–positive and auto-Ab–negative individuals (10.0% vs. 10.3%, P = 1.0).

**Table 4.** History of autoimmune disease, infection, and cancer among IFN-I auto-Ab–positive and –negative centenarians

Cohort	Characteristics	IFN-I auto-Ab- positive, *n*/*N* (%)	IFN-I auto-Ab- negative, *n*/*N* (%)	P value
Denmark	Autoimmune disease^a^	5/15 (33.3)	38/190 (20)	0.32
	Cancer history (including skin cancer)	5/15 (33.3)	34/195 (17.4)	0.16
Colombia	Autoimmune disease^a^	1/9 (11.1)	8/90 (8.9)	0.59
	Infection history (including COVID-19)	1/9 (11.1)	15/90 (16.7)	1
	Cancer history (including skin cancer)	2/9 (22.2)	4/90 (4.4)	0.09
Japan	Cancer history	1/10 (10.0)	29/282 (10.3%)	1

COVID-19, coronavirus disease 2019; n/N, number positive/number assessed. P values were calculated using two-tailed Fisher’s exact tests.

a Autoimmune disease in Danish centenarians includes rheumatoid arthritis, increased and decreased metabolism, and glaucoma. Autoimmune disease in Colombian centenarians includes rheumatoid arthritis, lupus, dry mucous membrane, hypothyroidism, hyperthyroidism, skin disease, and others.

### Mortality of Danish centenarians: A subanalysis

A subanalysis on mortality was conducted in the Danish centenarians to investigate potential differences in mortality between IFN-I auto-Ab–positive and IFN-I auto-Ab–negative Danish centenarians. Descriptive characteristics of the Danish centenarians, including lifestyle factors and follow-up information, are shown in Table S7. All centenarians died during follow-up. The mean and median survival time after blood sampling was 871 and 704 days, respectively. The 18 IFN-I auto-Ab–positive centenarians (14 females and 4 males) had a median survival from blood sampling that was 16 days shorter than that of IFN-I auto-Ab–negative centenarians (194 females and 46 males). The difference was not statistically significant (P = 0.77). In Cox regression models, no difference in mortality was observed for IFN-I auto-Ab–positive vs. –negative centenarians (unadjusted hazard ratio [HR], 1.08, 95% CI, 0.67–1.75; adjusted HR, 1.18, 95% CI, 0.72–1.93). For context, these HRs were smaller than the association between male vs. female mortality (unadjusted HR, 1.29, 95% CI, 0.95–1.76; adjusted HR, 1.28, 95% CI, 0.92–1.79). The proportional hazards assumption held across all models.

## Discussion

Here, we examined individuals at the extreme end of the human lifespan with the aim of clarifying whether the previously established age-associated increase in neutralizing IFN-I auto-Abs continues into advanced age and whether there is a difference in IFN-I auto-Abs prevalence across different ethnicities and geographical locations. In this multinational study of 938 centenarians and beyond from Denmark, Colombia, Australia, and Japan, we found a prevalence of 5.4% (51/938) centenarians having neutralizing IFN-I auto-Abs. Comparing the prevalence of neutralizing IFN-I auto-Abs in the centenarian cohorts to the prevalence in a large French general population cohort (6, 18) showed that the prevalence of IFN-I auto-Abs reaches a plateau with age rather than continuing to increase.

Recent studies have highlighted substantial interindividual variability in age-related thymic involution, demonstrating that thymic health varies with sex, age, and lifestyle factors, with females generally exhibiting better thymic health than males. Furthermore, individuals with poor thymic health display higher levels of systemic inflammation, increased risk of cardiovascular disease, cancer, and elevated all-cause mortality, as well as a reduced T cell receptor repertoire (33, 34). These findings support a role for thymic function in maintaining immune competence during aging and may contribute to the marked heterogeneity observed in healthy ageing and longevity. Although we did not directly assess thymic function or central tolerance, our findings are compatible with the hypothesis that mechanisms maintaining immune tolerance remain relatively preserved in individuals who achieve exceptional longevity, although declining with age in the majority of individuals. This interpretation is consistent with previous observations in Danish centenarians born in 1895, demonstrating preserved thyroid function compared with less old individuals (35). Moreover, although autoimmunity tends to increase with age and other circulating auto-Abs have been described in centenarians, clinical autoimmune disease in centenarians is rare (31, 36, 37). These observations may help explain why the age-associated increase in neutralizing IFN-I auto-Abs appears to level off rather than continue into extreme old age. A recent study found that a substantial proportion of Colombian centenarians had immune parameters comparable with those of less old individuals, while increased biological age was associated with markers of immune dysregulation (38), thereby adding to the existing knowledge regarding heterogeneity in immune function and regulation in centenarians (31). In this context, our results align well with the paradox of immune regulation seen in centenarians, where some individuals have markers of a relatively “young” immune system, while others have dysregulation and autoimmunity, including IFN-I auto-Abs.

In the present study, we did not observe any significant differences in IFN-I auto-Abs prevalence between sexes, either within the individual centenarian cohorts or in the combined analysis. Since all four centenarian cohorts were female-predominant (76.3% female), we additionally performed sex-stratified comparisons with the large French general population cohort (6, 18). Female centenarians exhibited a statistically significantly lower prevalence of IFN-I auto-Abs compared with females ≥80 years from the French general population cohort, whereas the prevalence between male centenarians and males ≥80 years in the general population was not statistically significantly different. When the clinical relevance of neutralizing IFN-I auto-Abs was first recognized, these were found predominantly in male patients with severe COVID-19 (5, 6). Subsequent studies of IFN-I auto-Abs have focused more on disease association rather than sex distribution, although higher prevalence of IFN-I auto-Abs was also seen in male individuals with critical influenza pneumonia (8) and WNV disease (9, 10). In contrast, the prevalence of IFN-I auto-Abs in the French general population cohort was more evenly distributed between sexes, with no difference in prevalence amongst males and females ≥80 years (6, 18), consistent with our observations in centenarians. Together, these findings suggest that male predominance in IFN-I auto-Abs prevalence may be more closely linked to susceptibility to severe viral disease rather than reflecting a characteristic of the French general population. It is well-established that females live longer than males (28) and, in this regard, our data indicate that females who do not harbor IFN-I auto-Abs are more likely to survive into extreme longevity, perhaps suggesting that the presence of IFN-I auto-Abs is one factor among many that negatively influences the chances of reaching very old age.

We observed a modest geographical variation in the prevalence of neutralizing IFN-I auto-Abs, ranging from 3.3% in Japanese centenarians to 9.1% in Colombian centenarians, although this is a numerical trend requiring further confirmation. Importantly, a significantly lower prevalence was observed in the Japanese centenarians compared with the French general population ≥80 years. During COVID-19, IFN-I auto-Abs were also investigated in the Japanese population, confirming a high prevalence among COVID-19 patients and a very low prevalence in the healthy Japanese control group (39). Indeed, it is well-established that Japan has the highest number of centenarians overall, and in 2024, around a fifth of the world’s centenarians were Japanese (28). In this context, it might not be surprising that the Japanese centenarians harbor the lowest prevalence of IFN-I auto-Abs. It could be speculated that the low IFN-I auto-Abs prevalence may contribute, among other factors, to the generally extensive longevity in this population, although more data on IFN-I auto-Abs prevalence in the Japanese general population is warranted to draw any firm conclusion on this issue. On the contrary, Colombian centenarians had the highest prevalence of IFN-I auto-Abs observed among the cohorts. The prevalence of IFN-I auto-Abs in the Danish and Australian centenarians is the most comparable with the prevalence in individuals ≥80 years in the French general population cohort, which may reflect more shared environmental, lifestyle, and healthcare-related factors, and maybe also more common genetic ancestry, across these countries. However, regardless of geography, we did not observe either a total disappearance or a significant increase of IFN-I auto-Abs in individual centenarian cohorts compared with individuals aged ≥80 years from a French population cohort. Collectively, these findings indicate that IFN-I auto-Abs emerge and persist consistently across geographically distinct populations and locations, including to some extent in very old individuals. This also implies that having IFN-I auto-Abs does not seem to be incompatible with longevity at the level of the individual, although it certainly increases susceptibility and mortality from severe viral infections (5, 6, 7, 8, 9, 10, 11, 12, 13, 14, 15, 16, 17).

A number of questions regarding the clinical relevance and impact of harboring IFN-I auto-Abs in centenarians remain. Longitudinal samples were not available for these cohorts, and it therefore remains unclear whether the IFN-I auto-Abs detected in centenarian samples had been present for years or had developed only briefly before blood sampling. Additionally, the plateau in IFN-I auto-Abs prevalence observed in this study may reflect survival bias, by which individuals aged ≥80 years carrying IFN-I auto-Abs are at increased risk of mortality from severe infections or cancer and therefore less likely to survive into centenarian age. The subanalysis on mortality in the Danish centenarians showed a slight and nonsignificant increase in mortality in IFN-I auto-Ab–positive individuals, which argues against survival bias as the only explanation for the observed plateau in IFN-I auto-Abs prevalence in this study. Other explanations contributing to the plateau could be immune exhaustion and immunosenescence in the centenarians (28, 38), associated with a general reduced immune reactivity and impaired ability to produce IFN-I auto-Abs, therefore accounting for a lower net influx of IFN-I auto-Ab–positive individuals in the oldest old. Similar scenarios with a plateau in prevalence in the oldest individuals have been observed in a cross-sectional study of mosaic loss of the Y (mLOY) chromosome in Danish men aged 56–100 years, where the prevalence of mLOY increased with age until age 85, after which it plateaued at around 40% (40), and in a study on DNA methylation age (DNAmAge) in older Danish twins, where DNAmAge was found to increase with age but plateaued as people got older (41). Similarly, regarding cancer in the oldest old, mortality rates reach a plateau, or sometimes even decrease, after ∼85 years of age depending on cancer type (28). Moreover, the prevalence of IFN-I auto-Abs in the general populations of Denmark, Colombia, Australia, and Japan is not currently available, for which reason we compared centenarians with the best available large general population data from France, assumed to be relatively representative of the general population, potentially conferring a limitation of our conclusions regarding geographical differences. Moreover, the observed plateau of IFN-I auto-Abs prevalence may also be influenced by factors other than age, such as geographical and ethnic background, cohort characteristics, and potential pre-analytical sample variability. Although the available clinical information revealed no significant associations between IFN-I auto-Abs and histories of autoimmune disease, infection, or cancer, these analyses were limited by the small number of auto-Ab–positive individuals and incomplete clinical data sets across cohorts. For future perspectives, studies should investigate IFN-I auto-Abs in longitudinal samples from older individuals over decades, alongside their association with morbidity, to elucidate the patterns of emergence and persistence of IFN-I auto-Abs and their potential role in disease development and mortality.

In conclusion, this centenarian study demonstrates that the rise in prevalence of neutralizing IFN-I auto-Abs reaches a plateau at the extreme end of human lifespan, and even significantly decreases in Japanese centenarians as well as in female centenarians across different geographical cohorts compared with the general population ≥80 years. This corroborates other immunological and demographic observations from studies on centenarians, together with findings on immune function and thymic health in old age and the impact of these immune parameters on morbidity and mortality. Overall, the presence of neutralizing IFN-I auto-Abs appears compatible with exceptional longevity, while the plateau in the prevalence could suggest that harboring IFN-I auto-Abs is associated with an increased overall mortality and that a very high prevalence in the population may not be beneficial for reaching exceptional longevity.

## Materials and methods

### Cohorts and samples

This multinational study consisted of cohorts of nonagenarians (defined as age 90–99 years), centenarians (defined as persons who have reached the age of 100 years), semi-supercentenarians (defined as age 105–109 years), and supercentenarians (defined as age ≥110 years), collectively referred to as centenarians throughout this paper. All individuals in our study were ≥95 years.

Plasma or serum samples were obtained from 938 individuals aged ≥95 years from four independent cohorts representing distinct geographic regions: Denmark (*n* = 258), Colombia (*n* = 99), Australia (*n* = 281), and Japan (*n* = 300). Age eligibility criteria were defined locally within each cohort (see below).

Danish centenarian cohorts: Plasma samples from 258 Danish individuals aged ≥99 years at sampling were obtained through the Danish Aging Research Center (42). Participants originated from the 1905 birth cohort (*n* = 126) with samples collected in 2005 and the 1915 birth cohort (*n* = 132) with samples collected in 2015. Overall, 208 individuals (80.6%) were female and 50 (19.4%) were male. Samples were retrieved from an established biobank.

Colombian centenarian cohort: Serum samples were obtained from 99 Colombian individuals aged ≥99 years at sampling through the Colombian Centenarians Alliance, Colombia (https://longevidadsaludable.com.co). The samples were collected as part of an ongoing study initiated in September 2023 (43). The cohort comprised 72 females (72.7%) and 27 males (27.3%).

Australian centenarian cohort: Serum samples were obtained from 281 Australian individuals aged ≥95 years at sampling through the Centre for Healthy Brain Ageing (CHeBA), Discipline of Psychiatry & Mental Health, School of Clinical Medicine, Faculty of Medicine and Health, UNSW Sydney, Australia. The samples were collected as part of an ongoing project, the Sydney Centenarian Study (44), and were collected between October 2009 and March 2020. The cohort comprised 184 females (65.5%) and 97 males (34.5%).

Japanese centenarian cohort: Serum samples were obtained from 300 Japanese individuals ≥100 years at sampling. These samples were randomly selected from 1,027 samples collected between 2000 and 2024 through two prospective cohort studies: the Tokyo Centenarian Study and the Japanese Semi-supercentenarian Study (45, 46). The cohort comprised 252 females (84.0%) and 48 males (16.0%).

All samples were stored at −80°C until analysis. Prior to functional assessment in the HEK cell–based neutralization assay (see below), samples were heat-inactivated at 56°C for 20 min.

The French general population cohort comprised 34,159 French individuals aged 20–100 years with an equal distribution of males and females (6). A subset of this cohort (*n* = 9,126) was analyzed for neutralizing auto-Abs against both IFN-β at 10 ng/ml and IFN-α2 and IFN-ω at 100 pg/ml (6, 18). We used data from this subgroup for our comparison analyses, as the centenarian cohorts were assessed using the same IFN-I subtypes and neutralization thresholds. For the additional analysis comparing prevalence of IFN-α2 and IFN-ω (100 pg/ml) in the centenarian cohorts to age groups 80–84, 85–89, and 90–110 years in the French general population cohort, a subgroup of 10,778 French individuals was used (6).

### Luciferase-based neutralization assay

Neutralizing activity against type IFN-I was assessed in Danish, Colombian, and Australian centenarians using a previously established HEK-293T cell–based luciferase reporter assay (6, 9). HEK-293T cells were seeded at 1.5 × 10^4^ cells per well in 96-well plates in DMEM (#D6429; Sigma-Aldrich) supplemented with 10% FCS (#F7524; Sigma-Aldrich) and 1% penicillin-streptomycin (100 IU/ml; #10378-016; Thermo Fisher Scientific). After 24 h, cells were transiently transfected with 98 ng of an IFN-stimulated response element-driven firefly luciferase reporter plasmid (pGL4.45) and 2 ng of a constitutive Renilla luciferase control plasmid per well using X-tremeGENE 9 DNA transfectant reagent (#06365787001; Sigma-Aldrich) at 3:1 reagent-to-DNA ratio in OptiMEM (#31-985-070; Thermo Fischer Scientific). 24 hours after transfection, culture medium was replaced with 2% FCS-containing DMEM supplemented with recombinant IFN-I at the indicated concentrations and 10% heat-inactivated patient plasma or serum. Cells were incubated for 16 h prior to luciferase quantification. Unglycosylated IFN-α2 and IFN-ω were tested at 10 ng/ml and 100 pg/ml (130-093-874; Miltenyi Biotec and 300-02j-100UG; PeproTech). Glycosylated IFN-β was tested at 10 ng/ml (300-02BC-100UG; PeproTech). Luciferase activity was measured using the Dual-Luciferase Reporter Assay System (#E2980; Promega) on a Synergy LX multimode plate reader (BioTek). Relative luciferase activity (RLA) was calculated (firefly luciferase activity divided by Renilla luciferase activity). To remove the baseline signal, RLA values were further normalized to the RLA value of a control sample from the same plate not stimulated with IFN-I. This baseline-corrected RLA value was then normalized to the median induction level of the plate of non-neutralizing samples. The results are expressed as a percentage of the normalized RLA after baseline correction, % (nRLA ratio). Samples were considered neutralizing if the RLA ratio was below 15% of the median control value from the same experiment. The 15% cutoff was selected based on prior validation studies demonstrating robust discrimination between neutralizing and non-neutralizing samples (6). All samples were tested once for each condition. For each plate, serum from two healthy adult blood donors was also collected to serve as negative controls. A sample from a patient with an adverse reaction to live attenuated yellow fever vaccine known to have broad neutralization status against all IFN-I conditions tested was used as a positive control.

Neutralizing activity against IFN-I was assessed in Japanese centenarians using the same protocol and stimulating with the same IFN-I conditions and concentrations as mentioned above. Serum samples from healthy donors were used as negative controls. For the positive controls, serum from a patient with chronic mucocutaneous candidiasis due to STAT1 gain-of-function was used for IFN-α2 and IFN-ω auto-Abs, and serum from a female centenarian with known neutralizing activity was used for IFN-β.

The same protocol was used to analyze all four centenarian cohorts, though the Japanese cohort was analyzed separately using other controls. In all four cohorts, negative and positive controls showed consistency in neutralization activity as shown in Fig. 2, B, D, F, and H.

### Clinical data from questionnaires

From the two Danish Centenarian cohorts, self-reported diseases were collected as part of larger questionnaires (1905 in 2005 and 1915 in 2015). These were collected in combination with collecting plasma samples. Participants were asked, “Did a doctor ever tell you that you have/had any of the following diseases?” From the questionnaires, we included questions on history of cancer (including skin cancer) and different autoimmune diseases (rheumatoid arthritis, increased metabolism [hyperthyroidism] or decreased metabolism [hypothyroidism], and glaucoma) in our analysis. For Danish centenarians, questionnaire information was available from 224/258 individuals, including 17 individuals positive for neutralizing IFN-I auto-Abs. Of these 224 individuals, 14/224 did not answer questions on cancer history and 19/224 did not answer all questions on autoimmune diseases. Information on body mass index (BMI), smoking, alcohol consumption, and education used for subanalysis of mortality of the Danish centenarians was also obtained from the questionnaires. All Danish centenarians were dead at the end of follow-up on August 1, 2024, and birth date and date of death were obtained through the Danish Civil Registration System (47).

Clinical data from the Colombian centenarians were collected through a semi-structured questionnaire administered during a medical interview with the centenarian and their caregiver, as reported elsewhere (43, 48). In our analysis, we included history of cancer (including skin cancer), history of infection (COVID-19 and lung infection other than COVID-19), and history of autoimmune disease (thyroid disease [both hypo- and hyperthyroidism], rheumatoid arthritis, lupus, dry mucous membrane, skin disease, and others).

Clinical data from Japanese centenarians were collected through medical interviews and physical examinations performed by trained geriatricians according to previously described protocols (46).

### Statistics

Prevalence of neutralizing IFN-I auto-Abs was calculated as the proportion of positive individuals within each cohort. Differences in prevalence between cohorts were assessed using Pearson’s chi-squared test on contingency tables comparing auto-Ab–positive and auto-Ab–negative individuals across cohorts. ORs and corresponding P values for the association between neutralizing IFN-I auto-Abs and centenarians were calculated relative to the general population for different age groups. Comparisons of proportions between groups were performed using the chi-square test without Yates’ correction when expected counts were >5 or Fisher’s exact test when counts were ≤5. CIs for prevalence estimates in the centenarian cohorts and the general population were calculated using binomial proportion CIs based on the Wilson method.

Subanalysis of the Danish centenarians: Descriptive survival measures, including median survival differences and maximum observed survival times since blood sampling, were calculated for IFN-I auto-Ab–positive and –negative males and females. Differences in survival time distributions between IFN-I auto-Ab–positive and –negative centenarians were assessed using the exact Wilcoxon rank-sum test. All-cause mortality for IFN-I auto-Ab–positive vs. –negative Danish centenarians was analyzed using Cox proportional hazards regression with age as the underlying time scale and delayed entry at the age of blood sampling. Unadjusted and adjusted models were fitted. Adjusted models included the following variables: sex, BMI, smoking (ever vs. never), alcohol consumption (ever vs. never), and education. Additionally, HRs for male vs. female mortality were estimated to provide context for the magnitude of the IFN-related HRs. These sex-specific HRs were estimated using similar Cox regression models with adjustment for the same variables except sex. Proportional hazards assumptions were assessed for all Cox models using Schoenfeld residuals.

Statistical analyses were conducted using Stata 19.5 or R (RStudio) (version 2026.01.2, Posit Software, PBC) or GraphPad Prism (version 11.0.0, GraphPad Software). Figures were generated using GraphPad Prism or Adobe Illustrator (Version 30.3, Adobe).

### Online supplemental material

Fig. S1 shows a sex-stratified comparison of IFN-I–neutralizing auto-Abs prevalence between centenarian cohorts and age-stratified individuals from the French general population. Fig. S2 shows a comparison of IFN-I–neutralizing auto-Abs prevalence between centenarian cohorts and age-stratified individuals from the French general population at high concentrations of IFN-α2 and IFN-ω. Fig. S3 shows a comparison of IFN-β–neutralizing auto-Abs prevalence between centenarian cohorts and age-stratified individuals from the French general population. Fig. S4 shows a comparison of IFN-I–neutralizing auto-Abs prevalence between centenarian cohorts and age-stratified individuals from the French general population under IFN-α and IFN-ω double-positive conditions. Fig. S5 shows prevalence of neutralizing IFN-I auto-Abs according to age within the centenarian cohorts. Table S1 shows a comparison of IFN-I auto-Abs prevalence between centenarian cohorts and age-stratified French population controls for the conditions: IFN-α and/or IFN-ω (100 pg/ml) and/or IFN-β (10 ng/ml). Table S2 shows a comparison of IFN-I auto-Abs prevalence between centenarian cohorts and age-stratified French population controls for the conditions: IFN-α and/or IFN-ω (100 pg/ml), divided into age groups of 80–84 years, 85–89 years, and 90–110 years. Table S3 shows a comparison of IFN-I auto-Abs prevalence between centenarian cohorts and age-stratified French population controls for the conditions: IFN-α and/or IFN-ω (100 pg/ml) and/or IFN-β (10 ng/ml), divided by gender. Table S4 shows a comparison of IFN-I auto-Abs prevalence between centenarian cohorts and age-stratified French population controls for the conditions: IFN-α (10 ng/ml); IFN-ω (10 ng/ml). Table S5 shows a comparison of IFN-I auto-Abs prevalence between centenarian cohorts and age-stratified French population controls for the condition: IFN-β (10 ng/ml). Table S6 shows a comparison of IFN-I auto-Abs prevalence between centenarian cohorts and age-stratified French population controls for the conditions: IFN-α and IFN-ω (10 ng/ml); IFN-α and IFN-ω (100 pg/ml). Table S7 shows characteristics of IFN-I auto-Ab–positive and –negative males and females for the Danish centenarians.

## Ethics statement

### Ethics approval

The current project has been approved by the Regional Committees on Health Research Ethics for Southern Denmark (project ID S-20230086) as well as by the Research & Innovation Organization at the University of Southern Denmark (registration number 12.067), which approves all scientific projects for the University of Southern Denmark according to the General Data Protection Regulation. The project uses already collected biological material from surveys of the 1905 cohort (ethical approval: VF-20040240) and the 1915 West cohort (ethical approval: S-20100011). The Australian Sydney Centenarian Study cohort was from CHeBA, Discipline of Psychiatry & Mental Health, School of Clinical Medicine, Faculty of Medicine and Health, UNSW Sydney, Australia, with ethics approval from the UNSW Sydney Human Research Ethics Committee (2007–2012: HREC07064; 2012–2017: HC12313; 2017–2022: HC17257; 2022–2027: HC220354). Participants consented to the collection and sharing of research data and biospecimen samples. The Colombian study was performed in compliance with Act 008430/1993 of the Ministry of Health of the Republic of Colombia, which classified it as minimal-risk research. The ethics committee of the Universidad de la Costa, Barranquilla, Colombia, approved this study. Informed consent was obtained from all the participants and their legal guardians. The Japanese centenarian cohort protocols were approved by the ethics committee of Keio University School of Medicine (ID: 20022020).

## Supplementary Material

Table S1 shows a comparison of IFN-I auto-Abs prevalence between centenarian cohorts and age-stratified French population controls for the conditions: IFN-α and/or IFN-ω (100 pg/ml) and/or IFN-β (10 ng/ml).

Table S2 shows a comparison of IFN-I auto-Abs prevalence between centenarian cohorts and age-stratified French population controls for the conditions: IFN-α and/or IFN-ω (100 pg/ml), divided into age groups of 80–84 years, 85–89 years, and 90–110 years.

Table S3 shows a comparison of IFN-I auto-Abs prevalence between centenarian cohorts and age-stratified French population controls for the conditions: IFN-α and/or IFN-ω (100 pg/ml) and/or IFN-β (10 ng/ml), divided by gender.

Table S4 shows a comparison of IFN-I auto-Abs prevalence between centenarian cohorts and age-stratified French population controls for the conditions: IFN-α (10 ng/ml); IFN-ω (10 ng/ml).

Table S5 shows a comparison of IFN-I auto-Abs prevalence between centenarian cohorts and age-stratified French population controls for the condition: IFN-β (10 ng/ml).

Table S6 shows a comparison of IFN-I auto-Abs prevalence between centenarian cohorts and age-stratified French population controls for the conditions: IFN-α and IFN-ω (10 ng/ml); IFN-α and IFN-ω (100 pg/ml).

Table S7 shows characteristics of IFN-I auto-Ab–positive and –negative males and females for the Danish centenarians.

## Data Availability

All data related to this study will be shared at the request of other investigators for purposes of replicating procedures and results, according to national and international General Data Protection Regulation rules, and following individual data transfer agreement and material transfer agreement rules with relevant investigators. Researchers wishing to apply for data from the Sydney Centenarian Study are directed to https://www.unsw.edu.au/cheba/dpau.
