# Supplementary material for: The rise in prevalence of autoantibodies neutralizing type I interferons reaches a plateau in centenarians
Source: J Hum Immun. 2026 Oct 27;3(1):e20260143. doi: 10.70962/jhi.20260143 (PMC13712330; doi:10.70962/jhi.20260143)
Supplement: Supplementary file 1 [file jhi_20260143_tables1.docx]

| **Analysis** | **Cohort** | **Comparison** | **OR** | **95% CI** | **p** |
| --- | --- | --- | --- | --- | --- |
| IFN-α and/or IFN-ω (100 pg/mL) and/or IFN-β (10 ng/mL) | Combined | <60 | 3.651 | 2.499-5.343 | <0.0001 |
| IFN-α and/or IFN-ω (100 pg/mL) and/or IFN-β (10 ng/mL) | Combined | 60-69 | 4.728 | 2.931-7.760 | <0.0001 |
| IFN-α and/or IFN-ω (100 pg/mL) and/or IFN-β (10 ng/mL) | Combined | 70-79 | 2.313 | 1.490-3.632 | 0.0001 |
| IFN-α and/or IFN-ω (100 pg/mL) and/or IFN-β (10 ng/mL) | Combined | ≥80 | 0.8607 | 0.6038-1.221 | 0.3993 |
| IFN-α and/or IFN-ω (100 pg/mL) and/or IFN-β (10 ng/mL) | Denmark | <60 | 4.763 | 2.805-8.117 | <0.0001 |
| IFN-α and/or IFN-ω (100 pg/mL) and/or IFN-β (10 ng/mL) | Denmark | 60-69 | 6.167 | 3.410-11.54 | <0.0001 |
| IFN-α and/or IFN-ω (100 pg/mL) and/or IFN-β (10 ng/mL) | Denmark | 70-79 | 3.017 | 1.651-5.311 | 0.0001 |
| IFN-α and/or IFN-ω (100 pg/mL) and/or IFN-β (10 ng/mL) | Denmark | ≥80 | 1.123 | 0.6796-1.882 | 0.6633 |
| IFN-α and/or IFN-ω (100 pg/mL) and/or IFN-β (10 ng/mL) | Colombia | <60 | 6.350 | 3.110-13.03 | <0.0001 |
| IFN-α and/or IFN-ω (100 pg/mL) and/or IFN-β (10 ng/mL) | Colombia | 60-69 | 8.223 | 3.680-17.82 | <0.0001 |
| IFN-α and/or IFN-ω (100 pg/mL) and/or IFN-β (10 ng/mL) | Colombia | 70-79 | 4.023 | 1.868-8.637 | 0.0001 |
| IFN-α and/or IFN-ω (100 pg/mL) and/or IFN-β (10 ng/mL) | Colombia | ≥80 | 1.497 | 0.7486-2.973 | 0.2660 |
| IFN-α and/or IFN-ω (100 pg/mL) and/or IFN-β (10 ng/mL) | Australia | <60 | 3.330 | 1.784-5.943 | <0.0001 |
| IFN-α and/or IFN-ω (100 pg/mL) and/or IFN-β (10 ng/mL) | Australia | 60-69 | 4.312 | 2.256-8.201 | <0.0001 |
| IFN-α and/or IFN-ω (100 pg/mL) and/or IFN-β (10 ng/mL) | Australia | 70-79 | 2.109 | 1.152-3.895 | 0.0183 |
| IFN-α and/or IFN-ω (100 pg/mL) and/or IFN-β (10 ng/mL) | Australia | ≥80 | 0.7849 | 0.4321-1.386 | 0.4083 |
| IFN-α and/or IFN-ω (100 pg/mL) and/or IFN-β (10 ng/mL) | Japan | <60 | 2.190 | 1.069-4.262 | 0.0200 |
| IFN-α and/or IFN-ω (100 pg/mL) and/or IFN-β (10 ng/mL) | Japan | 60-69 | 2.836 | 1.388-5.704 | 0.0039 |
| IFN-α and/or IFN-ω (100 pg/mL) and/or IFN-β (10 ng/mL) | Japan | 70-79 | 1.387 | 0.6786-2.737 | 0.3670 |
| IFN-α and/or IFN-ω (100 pg/mL) and/or IFN-β (10 ng/mL) | Japan | ≥80 | 0.6162 | 0.2578-1.002 | 0.0468 |

Supplementary Table 1. Comparison of IFN-I auto-Abs prevalence between centenarian cohorts and age-stratified French population controls for the condition: IFN-α and/or IFN-ω (100 pg/mL) and/or IFN-β (10 ng/mL)
