# Supplementary material for: The rise in prevalence of autoantibodies neutralizing type I interferons reaches a plateau in centenarians
Source: J Hum Immun. 2026 Oct 27;3(1):e20260143. doi: 10.70962/jhi.20260143 (PMC13712330; doi:10.70962/jhi.20260143)
Supplement: Supplementary file 2 [file jhi_20260143_tables2.docx]

| **Analysis** | **Cohort** | **Comparison** | **OR** | **95% CI** | **p** |
| --- | --- | --- | --- | --- | --- |
| IFN-α and/or IFN-ω (100 pg/mL) | Combined | 80-84 | 0.7320 | 0.4870-1.099 | 0.1277 |
| IFN-α and/or IFN-ω (100 pg/mL) | Combined | 85-89 | 1.027 | 0.6870-1.549 | 0.8986 |
| IFN-α and/or IFN-ω (100 pg/mL) | Combined | 90-110 | 0.7502 | 0.4844-1.171 | 0.2094 |
| IFN-α and/or IFN-ω (100 pg/mL) | Denmark | 80-84 | 0.9750 | 0.5576-1.686 | 0.9290 |
| IFN-α and/or IFN-ω (100 pg/mL) | Denmark | 85-89 | 1.368 | 0.7725-2.416 | 0.2751 |
| IFN-α and/or IFN-ω (100 pg/mL) | Denmark | 90-110 | 0.9993 | 0.5398-1.826 | 0.9981 |
| IFN-α and/or IFN-ω (100 pg/mL) | Colombia | 80-84 | 1.300 | 0.6237-2.640 | 0.4872 |
| IFN-α and/or IFN-ω (100 pg/mL) | Colombia | 85-89 | 1.824 | 0.8670-3.762 | 0.1102 |
| IFN-α and/or IFN-ω (100 pg/mL) | Colombia | 90-110 | 1.332 | 0.6133-2.747 | 0.4631 |
| IFN-α and/or IFN-ω (100 pg/mL) | Australia | 80-84 | 0.6816 | 0.3737-1.226 | 0.2144 |
| IFN-α and/or IFN-ω (100 pg/mL) | Australia | 85-89 | 0.9566 | 0.5257-1.754 | 0.8877 |
| IFN-α and/or IFN-ω (100 pg/mL) | Australia | 90-110 | 0.6986 | 0.3776-1.312 | 0.2702 |
| IFN-α and/or IFN-ω (100 pg/mL) | Japan | 80-84 | 0.4021 | 0.1994-0.8150 | 0.0107 |
| IFN-α and/or IFN-ω (100 pg/mL) | Japan | 85-89 | 0.5643 | 0.2772-1.164 | 0.1188 |
| IFN-α and/or IFN-ω (100 pg/mL) | Japan | 90-110 | 0.4021 | 0.1994-0.8150 | 0.0107 |

Supplementary Table 2. Comparison of IFN-I auto-Abs prevalence between centenarian cohorts and age-stratified French population controls for the conditions: IFN-α and/or IFN-ω (100 pg/mL), divided into age groups of 80-84 years, 85-89 years, and 90-110 years
