# Supplementary material for: The rise in prevalence of autoantibodies neutralizing type I interferons reaches a plateau in centenarians
Source: J Hum Immun. 2026 Oct 27;3(1):e20260143. doi: 10.70962/jhi.20260143 (PMC13712330; doi:10.70962/jhi.20260143)
Supplement: Supplementary file 3 [file jhi_20260143_tables3.docx]

| **Analysis** | **Cohort** | **Comparison** | **OR** | **95% CI** | **p** |
| --- | --- | --- | --- | --- | --- |
| IFN-α and/or IFN-ω (100 pg/mL) and/or IFN-β (10 ng/mL) for females | Combined | <60 | 3.158 | 1.990-5.021 | <0.0001 |
| IFN-α and/or IFN-ω (100 pg/mL) and/or IFN-β (10 ng/mL) for females | Combined | 60-69 | 5.689 | 2.809-11.69 | <0.0001 |
| IFN-α and/or IFN-ω (100 pg/mL) and/or IFN-β (10 ng/mL) for females | Combined | 70-79 | 3.917 | 1.940-8.067 | <0.0001 |
| IFN-α and/or IFN-ω (100 pg/mL) and/or IFN-β (10 ng/mL) for females | Combined | ≥80 | 0.6494 | 0.4267-0.9962 | 0.0440 |
| IFN-α and/or IFN-ω (100 pg/mL) and/or IFN-β (10 ng/mL) for females | Denmark | <60 | 4.182 | 2.278-7.729 | <0.0001 |
| IFN-α and/or IFN-ω (100 pg/mL) and/or IFN-β (10 ng/mL) for females | Denmark | 60-69 | 7.534 | 3.414-16.68 | <0.0001 |
| IFN-α and/or IFN-ω (100 pg/mL) and/or IFN-β (10 ng/mL) for females | Denmark | 70-79 | 5.188 | 2.267-12.67 | <0.0001 |
| IFN-α and/or IFN-ω (100 pg/mL) and/or IFN-β (10 ng/mL) for females | Denmark | ≥80 | 0.860 | 0.4699-1.579 | 0.6236 |
| IFN-α and/or IFN-ω (100 pg/mL) and/or IFN-β (10 ng/mL) for females | Colombia | <60 | 6.240 | 2.555-14.27 | <0.0001 |
| IFN-α and/or IFN-ω (100 pg/mL) and/or IFN-β (10 ng/mL) for females | Colombia | 60-69 | 11.24 | 4.309-31.73 | <0.0001 |
| IFN-α and/or IFN-ω (100 pg/mL) and/or IFN-β (10 ng/mL) for females | Colombia | 70-79 | 7.742 | 2.944-20.14 | <0.0001 |
| IFN-α and/or IFN-ω (100 pg/mL) and/or IFN-β (10 ng/mL) for females | Colombia | ≥80 | 1.283 | 0.5455-2.912 | 0.5515 |
| IFN-α and/or IFN-ω (100 pg/mL) and/or IFN-β (10 ng/mL) for females | Australia | <60 | 2.292 | 0.9737-4.970 | 0.0426 |
| IFN-α and/or IFN-ω (100 pg/mL) and/or IFN-β (10 ng/mL) for females | Australia | 60-69 | 4.129 | 1.587-11.24 | 0.0021 |
| IFN-α and/or IFN-ω (100 pg/mL) and/or IFN-β (10 ng/mL) for females | Australia | 70-79 | 2.843 | 1.087-7.175 | 0.0329 |
| IFN-α and/or IFN-ω (100 pg/mL) and/or IFN-β (10 ng/mL) for females | Australia | ≥80 | 0.4713 | 0.2079-1.009 | 0.0596 |
| IFN-α and/or IFN-ω (100 pg/mL) and/or IFN-β (10 ng/mL) for females | Japan | <60 | 2.069 | 0.9948-4.231 | 0.0498 |
| IFN-α and/or IFN-ω (100 pg/mL) and/or IFN-β (10 ng/mL) for females | Japan | 60-69 | 3.729 | 1.526-9.265 | 0.0024 |
| IFN-α and/or IFN-ω (100 pg/mL) and/or IFN-β (10 ng/mL) for females | Japan | 70-79 | 2.567 | 1.053-6.241 | 0.0408 |
| IFN-α and/or IFN-ω (100 pg/mL) and/or IFN-β (10 ng/mL) for females | Japan | ≥80 | 0.4256 | 0.2132-0.8471 | 0.0161 |
| IFN-α and/or IFN-ω (100 pg/mL) and/or IFN-β (10 ng/mL) for males | Combined | <60 | 4.755 | 2.503-8.908 | <0.0001 |
| IFN-α and/or IFN-ω (100 pg/mL) and/or IFN-β (10 ng/mL) for males | Combined | 60-69 | 4.493 | 2.113-9.145 | <0.0001 |
| IFN-α and/or IFN-ω (100 pg/mL) and/or IFN-β (10 ng/mL) for males | Combined | 70-79 | 1.879 | 0.9736-3.618 | 0.0602 |
| IFN-α and/or IFN-ω (100 pg/mL) and/or IFN-β (10 ng/mL) for males | Combined | ≥80 | 1.164 | 0.6344-2.151 | 0.6383 |
| IFN-α and/or IFN-ω (100 pg/mL) and/or IFN-β (10 ng/mL) for males | Denmark | <60 | 6.143 | 2.234-17.07 | 0.0067 |
| IFN-α and/or IFN-ω (100 pg/mL) and/or IFN-β (10 ng/mL) (Males only) | Denmark | 60-69 | 5.804 | 2.032-16.82 | 0.0096 |
| IFN-α and/or IFN-ω (100 pg/mL) and/or IFN-β (10 ng/mL) for males | Denmark | 70-79 | 2.428 | 0.8789-6.871 | 0.1102 |
| IFN-α and/or IFN-ω (100 pg/mL) and/or IFN-β (10 ng/mL) for males | Denmark | ≥80 | 1.504 | 0.5535-4.026 | 0.5175 |
| IFN-α and/or IFN-ω (100 pg/mL) and/or IFN-β (10 ng/mL) for males | Colombia | <60 | 5.651 | 1.265-23.03 | 0.0588 |
| IFN-α and/or IFN-ω (100 pg/mL) and/or IFN-β (10 ng/mL) for males | Colombia | 60-69 | 5.340 | 1.161-21.48 | 0.0685 |
| IFN-α and/or IFN-ω (100 pg/mL) and/or IFN-β (10 ng/mL) for males | Colombia | 70-79 | 2.234 | 0.4984-9.249 | 0.2627 |
| IFN-α and/or IFN-ω (100 pg/mL) and/or IFN-β (10 ng/mL) for males | Colombia | ≥80 | 1.384 | 0.3127-5.465 | 0.6567 |
| IFN-α and/or IFN-ω (100 pg/mL) and/or IFN-β (10 ng/mL) for males | Australia | <60 | 5.494 | 2.224-12.92 | <0.0001 |
| IFN-α and/or IFN-ω (100 pg/mL) and/or IFN-β (10 ng/mL) for males | Australia | 60-69 | 5.192 | 1.957-12.48 | <0.0001 |
| IFN-α and/or IFN-ω (100 pg/mL) and/or IFN-β (10 ng/mL) for males | Australia | 70-79 | 2.172 | 0.8687-4.885 | 0.0714 |
| IFN-α and/or IFN-ω (100 pg/mL) and/or IFN-β (10 ng/mL) for males | Australia | ≥80 | 1.345 | 0.5594-3.009 | 0.4860 |
| IFN-α and/or IFN-ω (100 pg/mL) and/or IFN-β (10 ng/mL) for males | Japan | <60 | 1.503 | 0.1431-8.232 | 0.4987 |
| IFN-α and/or IFN-ω (100 pg/mL) and/or IFN-β (10 ng/mL) for males | Japan | 60-69 | 1.420 | 0.1321-8.638 | 0.5238 |
| IFN-α and/or IFN-ω (100 pg/mL) and/or IFN-β (10 ng/mL) for males | Japan | 70-79 | 0.5941 | 0.0564-3.297 | <0.999 |
| IFN-α and/or IFN-ω (100 pg/mL) and/or IFN-β (10 ng/mL) for males | Japan | ≥80 | 0.3680 | 0.0353-2.199 | 0.5042 |

Supplementary Table 3. Comparison of IFN-I auto-Abs prevalence between centenarian cohorts and age-stratified French population controls for the conditions: IFN-α and/or IFN-ω (100 pg/mL) and/or IFN-β (10 ng/mL), divided by gender
