# Supplementary material for: The rise in prevalence of autoantibodies neutralizing type I interferons reaches a plateau in centenarians
Source: J Hum Immun. 2026 Oct 27;3(1):e20260143. doi: 10.70962/jhi.20260143 (PMC13712330; doi:10.70962/jhi.20260143)
Supplement: Supplementary file 4 [file jhi_20260143_tables4.docx]

| **Analysis** | **Cohort** | **Comparison** | **OR** | **95% CI** | **p** |
| --- | --- | --- | --- | --- | --- |
| IFN-α (10 ng/mL) | Combined | <60 | 29.96 | 9.335-95.76 | <0.0001 |
| IFN-α (10 ng/mL) | Combined | 60-69 | 46.56 | 8.589-486.0 | <0.0001 |
| IFN-α (10 ng/mL) | Combined | 70-79 | 2.534 | 1.1245-5.047 | 0.0088 |
| IFN-α (10 ng/mL) | Combined | ≥80 | 0.9368 | 0.5468-1.625 | 0.8198 |
| IFN-α (10 ng/mL) | Denmark | <60 | 61.23 | 18.98-206.0 | <0.0001 |
| IFN-α (10 ng/mL) | Denmark | 60-69 | 95.17 | 14.46-1028 | <0.0001 |
| IFN-α (10 ng/mL) | Denmark | 70-79 | 5.181 | 2.343-12.05 | <0.0001 |
| IFN-α (10 ng/mL) | Denmark | ≥80 | 1.915 | 0.9588-3.711 | 0.0627 |
| IFN-α (10 ng/mL) | Colombia | <60 | 14.03 | 1.070-94.51 | 0.0905 |
| IFN-α (10 ng/mL) | Colombia | 60-69 | 21.81 | 1.137-414.3 | 0.0866 |
| IFN-α (10 ng/mL) | Colombia | 70-79 | 1.187 | 0.1096-7.646 | 0.5881 |
| IFN-α (10 ng/mL) | Colombia | ≥80 | 0.4388 | 0.0424-2.538 | 0.703 |
| IFN-α (10 ng/mL) | Australia | <60 | 24.91 | 6.607-94.34 | <0.0001 |
| IFN-α (10 ng/mL) | Australia | 60-69 | 38.71 | 5.348-456.5 | 0.0001 |
| IFN-α (10 ng/mL) | Australia | 70-79 | 2.107 | 0.8148-5.792 | 0.1824 |
| IFN-α (10 ng/mL) | Australia | ≥80 | 0.7790 | 0.3261-1.988 | 0.8236 |
| IFN-α (10 ng/mL) | Japan | <60 | 13.89 | 3.226-59.61 | 0.0053 |
| IFN-α (10 ng/mL) | Japan | 60-69 | 21.59 | 3.205-280.5 | 0.0067 |
| IFN-α (10 ng/mL) | Japan | 70-79 | 1.175 | 0.3515-4.160 | 0.7363 |
| IFN-α (10 ng/mL) | Japan | ≥80 | 0.4343 | 0.1388-1.271 | 0.1850 |
| IFN-ω (10 ng/mL) | Combined | <60 | 11.59 | 4.432-29.54 | <0.0001 |
| IFN-ω (10 ng/mL) | Combined | 60-69 | ∞ | 7.958-∞ | <0.0001 |
| IFN-ω (10 ng/mL) | Combined | 70-79 | 1.399 | 0.6835-3.074 | 0.3837 |
| IFN-ω (10 ng/mL) | Combined | ≥80 | 0.8022 | 0.4014-1.559 | 0.5216 |
| IFN-ω (10 ng/mL) | Denmark | <60 | 12.99 | 3.955-43.07 | 0.0012 |
| IFN-ω (10 ng/mL) | Denmark | 60-69 | ∞ | 8.330-∞ | 0.0001 |
| IFN-ω (10 ng/mL) | Denmark | 70-79 | 1.568 | 0.5574-4.457 | 0.5065 |
| IFN-ω (10 ng/mL) | Denmark | ≥80 | 0.8989 | 0.3337-2.557 | >0.9999 |
| IFN-ω (10 ng/mL) | Colombia | <60 | 17.00 | 3.338-79.85 | 0.0106 |
| IFN-ω (10 ng/mL) | Colombia | 60-69 | ∞ | 10.05-∞ | 0.0019 |
| IFN-ω (10 ng/mL) | Colombia | 70-79 | 2.053 | 0.4572-7.758 | 0.2835 |
| IFN-ω (10 ng/mL) | Colombia | ≥80 | 1.177 | 0.2711-4.340 | 0.6891 |
| IFN-ω (10 ng/mL) | Australia | <60 | 14.94 | 4.888-45.51 | 0.0002 |
| IFN-ω (10 ng/mL) | Australia | 60-69 | ∞ | 11.47-∞ | <0.0001 |
| IFN-ω (10 ng/mL) | Australia | 70-79 | 1.804 | 0.7093-4.696 | 0.2277 |
| IFN-ω (10 ng/mL) | Australia | ≥80 | 1.034 | 0.4263-2.622 | >0.999 |
| IFN-ω (10 ng/mL) | Japan | <60 | 5.535 | 1.095-25.63 | 0.0766 |
| IFN-ω (10 ng/mL) | Japan | 60-69 | ∞ | 3.303-∞ | 0.0151 |
| IFN-ω (10 ng/mL) | Japan | 70-79 | 0.6683 | 0.1500-2.471 | >0.999 |
| IFN-ω (10 ng/mL) | Japan | ≥80 | 0.3831 | 0.0889-1.513 | 0.2985 |

Supplementary Table 4. Comparison of IFN-I auto-Abs prevalence between centenarian cohorts and age-stratified French population controls for the conditions: IFN-α (10 ng/mL); IFN-ω (10 ng/mL)
