# Supplementary material for: The rise in prevalence of autoantibodies neutralizing type I interferons reaches a plateau in centenarians
Source: J Hum Immun. 2026 Oct 27;3(1):e20260143. doi: 10.70962/jhi.20260143 (PMC13712330; doi:10.70962/jhi.20260143)
Supplement: Supplementary file 5 [file jhi_20260143_tables5.docx]

| **Analysis** | **Cohort** | **Comparison** | **OR** | **95% CI** | **p** |
| --- | --- | --- | --- | --- | --- |
| IFN-β (10 ng/mL) | Combined | <60 | 1.892 | 0.7555-4.914 | 0.2415 |
| IFN-β (10 ng/mL) | Combined | 60-69 | 3.435 | 0.9346-10.81 | 0.0769 |
| IFN-β (10 ng/mL) | Combined | 70-79 | 1.504 | 0.4793-4.719 | 0.5591 |
| IFN-β (10 ng/mL) | Combined | ≥80 | ∞ | 2.219-∞ | 0.0036 |
| IFN-β (10 ng/mL) | Denmark | <60 | 2.296 | 0.5151-8.488 | 0.2417 |
| IFN-β (10 ng/mL) | Denmark | 60-69 | 4.168 | 0.7886-17.94 | 0.1295 |
| IFN-β (10 ng/mL) | Denmark | 70-79 | 1.826 | 0.3724-7.581 | 0.3583 |
| IFN-β (10 ng/mL) | Denmark | ≥80 | ∞ | 2.609-∞ | 0.0227 |
| IFN-β (10 ng/mL) | Colombia | <60 | 0.000 | 0.000-11.24 | >0.9999 |
| IFN-β (10 ng/mL) | Colombia | 60-69 | 0.000 | 0.000-21.91 | >0.9999 |
| IFN-β (10 ng/mL) | Colombia | 70-79 | 0.000 | 0.000-10.12 | >0.9999 |
| IFN-β (10 ng/mL) | Colombia | ≥80 | - | - | >0.9999 |
| IFN-β (10 ng/mL) | Australia | <60 | 2.107 | 0.4728-7.781 | 0.2716 |
| IFN-β (10 ng/mL) | Australia | 60-69 | 3.824 | 0.7239-16.45 | 0.1472 |
| IFN-β (10 ng/mL) | Australia | 70-79 | 1.675 | 0.3419-6.957 | 0.6270 |
| IFN-β (10 ng/mL) | Australia | ≥80 | ∞ | 2.395-∞ | 0.0262 |
| IFN-β (10 ng/mL) | Japan | <60 | 1.972 | 0.4428-7.280 | 0.2961 |
| IFN-β (10 ng/mL) | Japan | 60-69 | 3.581 | 0.6779-15.40 | 0.1621 |
| IFN-β (10 ng/mL) | Japan | 70-79 | 1.568 | 0.3201-6.514 | 0.6359 |
| IFN-β (10 ng/mL) | Japan | ≥80 | ∞ | 2.243-∞ | 0.0292 |

Supplementary Table 5. Comparison of IFN-I auto-Abs prevalence between centenarian cohorts and age-stratified French population controls for the condition: IFN-β (10 ng/mL)
