# Supplementary material for: The rise in prevalence of autoantibodies neutralizing type I interferons reaches a plateau in centenarians
Source: J Hum Immun. 2026 Oct 27;3(1):e20260143. doi: 10.70962/jhi.20260143 (PMC13712330; doi:10.70962/jhi.20260143)
Supplement: Supplementary file 6 [file jhi_20260143_tables6.docx]

| **Analysis** | **Cohort** | **Comparison** | **OR** | **95% CI** | **p** |
| --- | --- | --- | --- | --- | --- |
| IFN-α and IFN-ω (10 ng/mL) | Combined | <60 | ∞ | 9.892-∞ | <0.0001 |
| IFN-α and IFN-ω (10 ng/mL) | Combined | 60-69 | ∞ | 5.121-∞ | <0.0001 |
| IFN-α and IFN-ω (10 ng/mL) | Combined | 70-79 | 1.203 | 0.4904-3.070 | 0.6981 |
| IFN-α and IFN-ω (10 ng/mL) | Combined | ≥80 | 1.127 | 0.4712-2.690 | 0.7978 |
| IFN-α and IFN-ω (10 ng/mL) | Denmark | <60 | ∞ | 13.94-∞ | 0.0002 |
| IFN-α and IFN-ω (10 ng/mL) | Denmark | 60-69 | ∞ | 7.217-∞ | 0.0012 |
| IFN-α and IFN-ω (10 ng/mL) | Denmark | 70-79 | 1.645 | 0.4820-5.564 | 0.4377 |
| IFN-α and IFN-ω (10 ng/mL) | Denmark | ≥80 | 1.541 | 0.4565-4.963 | 0.4560 |
| IFN-α and IFN-ω (10 ng/mL) | Colombia | <60 | - | - | >0.9999 |
| IFN-α and IFN-ω (10 ng/mL) | Colombia | 60-69 | - | - | >0.9999 |
| IFN-α and IFN-ω (10 ng/mL) | Colombia | 70-79 | 0.000 | 0.000-5.958 | >0.9999 |
| IFN-α and IFN-ω (10 ng/mL) | Colombia | ≥80 | 0.000 | 0.000-5.385 | >0.9999 |
| IFN-α and IFN-ω (10 ng/mL) | Australia | <60 | ∞ | 12.79-∞ | 0.0003 |
| IFN-α and IFN-ω (10 ng/mL) | Australia | 60-69 | ∞ | 6.624-∞ | 0.0016 |
| IFN-α and IFN-ω (10 ng/mL) | Australia | 70-79 | 1.509 | 0.4423-5.098 | 0.4638 |
| IFN-α and IFN-ω (10 ng/mL) | Australia | ≥80 | 1.414 | 0.4189-4.548 | 0.4853 |
| IFN-α and IFN-ω (10 ng/mL) | Japan | <60 | ∞ | 6.377-∞ | 0.0046 |
| IFN-α and IFN-ω (10 ng/mL) | Japan | 60-69 | ∞ | 3.303-∞ | 0.0151 |
| IFN-α and IFN-ω (10 ng/mL) | Japan | 70-79 | 0.9383 | 0.2045-4.002 | >0.9999 |
| IFN-α and IFN-ω (10 ng/mL) | Japan | ≥80 | 0.8792 | 0.1934-3.569 | >0.9999 |
| IFN-α and IFN-ω (100 pg/mL) | Combined | <60 | ∞ | 19.94-∞ | <0.0001 |
| IFN-α and IFN-ω (100 pg/mL) | Combined | 60-69 | 18.53 | 4.668-81.51 | <0.0001 |
| IFN-α and IFN-ω (100 pg/mL) | Combined | 70-79 | 1.612 | 0.8206-3.220 | 0.1833 |
| IFN-α and IFN-ω (100 pg/mL) | Combined | ≥80 | 0.7462 | 0.4123-1.377 | 0.3395 |
| IFN-α and IFN-ω (100 pg/mL) | Denmark | <60 | ∞ | 23.15-∞ | <0.0001 |
| IFN-α and IFN-ω (100 pg/mL) | Denmark | 60-69 | 25.43 | 6.103-124.2 | <0.0001 |
| IFN-α and IFN-ω (100 pg/mL) | Denmark | 70-79 | 2.211 | 0.8845-5.637 | 0.0953 |
| IFN-α and IFN-ω (100 pg/mL) | Denmark | ≥80 | 1.024 | 0.4552-2.398 | 0.9582 |
| IFN-α and IFN-ω (100 pg/mL) | Colombia | <60 | ∞ | 36.66-∞ | <0.0001 |
| IFN-α and IFN-ω (100 pg/mL) | Colombia | 60-69 | 33.38 | 6.709-188.7 | 0.0008 |
| IFN-α and IFN-ω (100 pg/mL) | Colombia | 70-79 | 2.902 | 0.8771-9.560 | 0.1094 |
| IFN-α and IFN-ω (100 pg/mL) | Colombia | ≥80 | 1.344 | 0.4252-4.048 | 0.4972 |
| IFN-α and IFN-ω (100 pg/mL) | Australia | <60 | ∞ | 22.16-∞ | <0.0001 |
| IFN-α and IFN-ω (100 pg/mL) | Australia | 60-69 | 19.35 | 4.161-97.47 | 0.0004 |
| IFN-α and IFN-ω (100 pg/mL) | Australia | 70-79 | 1.682 | 0.6662-4.642 | 0.3587 |
| IFN-α and IFN-ω (100 pg/mL) | Australia | ≥80 | 0.7790 | 0.3261-1.988 | 0.8236 |
| IFN-α and IFN-ω (100 pg/mL) | Japan | <60 | ∞ | 6.377-∞ | 0.0046 |
| IFN-α and IFN-ω (100 pg/mL) | Japan | 60-69 | 7.168 | 1.117-45.85 | 0.0765 |
| IFN-α and IFN-ω (100 pg/mL) | Japan | 70-79 | 0.6233 | 0.1406-2.552 | 0.7521 |
| IFN-α and IFN-ω (100 pg/mL) | Japan | ≥80 | 0.2886 | 0.0677-1.086 | 0.0720 |

Supplementary Table 6. Comparison of IFN-I auto-Abs prevalence between centenarian cohorts and age-stratified French population controls for the conditions: IFN-α and IFN-ω (10 ng/mL); IFN-α and IFN-ω (100 pg/mL)
