# Supplementary material for: The rise in prevalence of autoantibodies neutralizing type I interferons reaches a plateau in centenarians
Source: J Hum Immun. 2026 Oct 27;3(1):e20260143. doi: 10.70962/jhi.20260143 (PMC13712330; doi:10.70962/jhi.20260143)
Supplement: Supplementary file 7 [file jhi_20260143_tables7.docx]

|  | **IFN-I auto-Abs positive males** | **IFN-I auto-Abs negative males** | **IFN-I auto-Abs positive females** | **IFN-I auto-Abs negative females** |
| --- | --- | --- | --- | --- |
| N | 4 | 46 | 14 | 194 |
| Age in years at blood sampling, median (range) | 99.9 (99.9-100.0) | 100.1 (99.2-100.3) | 100.0 (99.3-100.2) | 100.0 (99.3-100.4) |
| Total observed time, years | 10.1 | 90.3 | 30.3 | 484.9 |
| Median observed time (range) | 2.1 (0.8-5.0) | 1.7 (0.1-7.0) | 1.8 (0.2-5.9) | 2.0 (0.0-10.8) |
| BMI, mean (sd) | 26.0 (2.5) | 23.8 (4.3) | 19.3 (2.5)  (1 missing) | 22.3 (4.2) |
| Education^a^ (higher education), n (%) | 0 (0%) | 9 (20%) (1 missing) | 1 (93%) | 20 (10%) |
| Alcohol consumption (drinker), n (%) | 3 (75%) | 38 (83%) | 7 (50%) | 127 (65%) (2 missing) |
| Smoking (smoker), n (%) | 2 (50%) | 33 (72%) (1 missing) | 5 (36%) | 73 (38%) (2 missing) |

Supplementary Table 7. Characteristics of IFN-I auto-Abs positive and negative males and females for the Danish centenarians.

^a^Education was classified according to the International Standard Classification of Education (ISCED 2011) and included as a binary variable (higher education yes/no). Higher education comprised ISCED levels 5-7 (short-cycle tertiary education, bachelor’s, or master’s level), while lower education comprised levels 1-4 (primary to upper secondary education).
